# The snow alga *Chloromonas kaweckae* sp. nov. (Volvocales, Chlorophyta) causes green surface blooms in the high tatras (Slovakia) and tolerates high irradiance

**DOI:** 10.1111/jpy.13307

**Published:** 2023-01-13

**Authors:** Lenka Procházková, Ryo Matsuzaki, Tomáš Řezanka, Linda Nedbalová, Daniel Remias

**Affiliations:** ^1^ Department of Ecology Charles University, Faculty of Science Prague 128 44 Czech Republic; ^2^ The Czech Academy of Sciences, Institute of Botany, Centre for Phycology Dukelská 135 379 82 Třeboň Czech Republic; ^3^ University of Tsukuba, Faculty of Life and Environmental Sciences 1–1–1 Tennodai Tsukuba Ibaraki 305–8572 Japan; ^4^ National Institute for Environmental Studies, Biodiversity Division 16‐2 Onogawa Tsukuba Ibaraki 305‐8506 Japan; ^5^ The Czech Academy of Sciences Institute of Microbiology Vídeňská 1083 Prague 142 20 Czech Republic; ^6^ University of Applied Sciences Upper Austria, School of Engineering Stelzhamerstr. 23 Wels 4600 Austria

**Keywords:** biodiversity, cryoflora, environmental sample, fatty acids, fluorometry, vegetative stages

## Abstract

Seasonally slowly melting mountain snowfields are populated by extremophilic microalgae. In alpine habitats, high‐light sensitive, green phytoflagellates are usually observed in subsurface layers deeper in the snowpack under dim conditions, while robust orange to reddish cyst stages can be seen exposed on the surface. In this study, uncommon surface green snow was investigated in the High Tatra Mountains (Slovakia). The monospecific community found in the green surface bloom consisted of vegetative *Chloromonas* cells (Volvocales, Chlorophyta). Molecular data demonstrated that the field sample and the strain isolated and established from the bloom were conspecific, and they represent a new species, *Chloromonas kaweckae* sp. nov., which is described based on the morphology of the vegetative cells and asexual reproduction and on molecular analyses of the strain. Cells of *C. kaweckae* accumulated approximately 50% polyunsaturated fatty acids, which is advantageous at low temperatures. In addition, this new species performed active photosynthesis at temperatures close to the freezing point showed a light compensation point of 126 ± 22 μmol photons · m^−2^ · s^−1^ and some signs of photoinhibition at irradiances greater than 600 μmol photons · m^−2^ · s^−1^. These data indicate that the photosynthetic apparatus of *C. kaweckae* could be regarded as adapted to relatively high light intensities, otherwise unusual for most flagellate stages of snow algae.

**Abbreviations**
BBM: Bold's basal medium
BI: Bayesian inference
BV: bootstrap values
CBC: compensatory base change
CCCryo: Culture Collection of Cryophilic Algae
DDBJ: DNA DataBank of Japan
ENA: European Nucleotide Archive
FAME: fatty acid methyl esters
GTR: general time reversible
I_k_
: light saturation point
ML: maximum likelihood
MUFA: monounsaturated fatty acid
NCBI: National Center for Biotechnology Information
NIES‐Collection: Microbial Culture Collection at the National Institute for Environmental Studies
nt: nucleotide
PAM: pulse‐amplitude modulated fluorometry
PAR: photosynthetically active radiation
PFD: photon flux densities
P‐I curve: photosynthesis‐irradiance curve
PP: posterior probabilities
PSII: photosystem II
PUFA: polyunsaturated fatty acid
rETR: the relative electron transport rate
SAFA: saturated fatty acid
UTEX: Culture Collection of Algae at The University of Texas at Austin
α: slope in the light‐limited section of the photosynthesis‐irradiance curve

1

Blooms of snow algae are observed in polar and mountainous regions worldwide (Matsuzaki et al. 2019, Hoham and Remias 2020). Together with halophiles and thermophiles, these psychrophiles belong to the extremophilic microorganisms that can be found in Central Europe (Zgonik et al. 2021). In the present study, we describe a rare green surface bloom dominated by a new species in the High Tatra Mts. (Poland, Slovakia). This mountain range represents the highest part of the Carpathian Arc and it is rich in lakes of glacial origin, which are surrounded by moraine deposits.

Green algae of the Chlamydomonadaceae, Volvocales (or Chlamydomonadales), dominate the photosynthetic communities in mountain snow (Krug et al. 2020). They evolved complex life cycles which enabled them to colonize melting snow (Hoham and Duval 2001, Matsuzaki et al. 2020). At exposed sites, the apparently high‐light sensitive green flagellate stages are usually found in deeper parts of the snowpack under milder light conditions, while later in the season, when the cells are exposed on the snow surface, they already have entered robust and orange to reddish pigmented cyst stages that are less susceptible to damage from high irradiance and other stresses (Sattler et al. 2012). At shaded sites under tree canopies, green snow spots made of *Chloromonas* were reported from the uppermost 5–15 or 20 cm of melting snow (e.g., *Chloromonas tughillensis* and *Chloromonas chenangoensis*; Hoham et al. 2006), or reached the snow surface (*Chloromonas hohamii*, Hoham et al. 1983; *Chloromonas* spp., Nedbalová et al. 2008; the summit plateau of Ruapehu, New Zealand, P. Novis, pers. comm.). An exception from the rule represents polar regions, where *Chloromonas* green blooms were clearly apparent on snow surfaces at exposed sites (e.g., Davey et al. 2019).

In the High Tatra Mts. (and likely in the majority of mid‐latitude mountain ranges), cysts of snow algae usually cause either surface orange or reddish snow (Kol 1968, Kawecka 1981, Procházková et al. 2018a,b, 2019a, 2020). Surface green snow caused by vegetative stages has been reported so far only from a semi‐shaded site, caused by the trebouxiophycean alga *Koliella tatrae* (L. Kováčik, pers. comm.; Hindák and Komárek 1968) or from a site above timberline, caused by *Carteria győrffyi* (Kol 1968, 1975). The first report of rare green alpine snow in the High Tatras (from Handel Valley above Zelené Krivánske lake) was already made by Czirbesz (1772). Similarly, surface green snow at exposed sites was observed by the authors during the melting season in several valleys of this mountain range. Since this phenomenon was absent in any other literature for nonpolar regions, the causative organism of the bloom was studied.

The main aim of this study was to identify and characterize the new snow alga. Analysis of multiple molecular markers indicated that the bloom represents a new species that was described here as *Chloromonas kaweckae* Procházková & Matsuzaki sp. nov., based on characteristics of the vegetative cell and asexual reproduction. Chlorophyll fluorometry revealed the high‐light adaptation of photosystem II (PSII), and this was complemented by a fatty acid profile rich in polyunsaturated fatty acids, as the rate of desaturation of these compounds is regarded as important for adaptation to low temperature (Morales‐Sánchez et al. 2020). Marker sequences of this species were found in several previously examined metagenomic datasets from Asia and other parts of Europe, excluding any hypothesis that this *Chloromonas* responsible for surface green snow is endemic.

## 2 MATERIALS AND METHODS

The green cryoflora was collected on 16 June 2018 in the upper part of Malá Studená Valley, High Tatras, Slovakia (ID of field sample = WP187). Global positioning system location and altitude were N 49°11.515′, E 20°11.628′, and 2026 m a. s. l., respectively. A thin layer of approximately 0.5 cm surface snow was removed with a sterile stainless‐steel scoop to reduce the content of dark snow detritus. With a field microscope, a virtually monospecific algal bloom was observed, and the cells were harvested down to an approximate depth of 3 cm into 50 mL sterile plastic centrifugation tubes. Additionally, a 10 mL subsample of WP187 was fixed immediately by a drop of acidic Lugol's solution (10% dilute acetic acid). Prior to further steps, snow was gently melted at 4°C in the dark. A unialgal strain was established by picking cell colonies out of field sample WP187 with a sterile loop on agar plates (Bold's basal medium [BBM], pH 6.4), grown at 5°C at approximately 20 μmol photons · m^−2^ · s^−1^. The newly established lab strain was then purified using the pipette‐washing method (Pringsheim 1946). The clonal and axenic strain was deposited as NIES‐4476 in the Microbial Culture Collection at the National Institute for Environmental Studies, Japan (https://mcc.nies.go.jp/index_en.html). The strain was maintained on an AF‐6 medium (https://mcc.nies.go.jp/02medium‐e.html) with a light:dark cycle of 14:10 h at 35–90 μmol photons · m^−2^ · s^−1^, as described previously (Matsuzaki et al. 2019).

The electrical conductivity and pH of the meltwater were measured immediately after melting with a conductometer (WTW Instruments, Weilheim, Germany). Light microscopy (LM) was performed with an Olympus BX43 (Olympus Corporation, Japan) equipped with a digital camera DXM 1200F (Nikon, USA) for the field sample and conducted with an Olympus BX51 equipped with Nomarski differential interference optics and an Olympus DP72 digital camera (Olympus) for the strain NIES‐4476. The protocol described in Procházková et al. (2018b) was used to determine maximal algal cell concentration per snow meltwater volume.

Using the temperature threshold of 15°C for optimal growth, one can separate the psychrophilic algae from the psychrotrophic ones (Leya 2013). To briefly check this assignment for the studied microorganism, the Erlenmeyer flask with strain NIES‐4476 was placed in BBM at 15°C. The growth was visually inspected after 2 weeks.

Total genomic DNA was extracted from the field material (WP187) and the strain NIES‐4476 according to Procházková et al. (2018b) and Nakada et al. (2008), respectively. Amplification and sequencing reactions of the internal transcribed spacer region 2 of ribosomal DNA (ITS2) for the field sample WP187 were identical to those described by Procházková et al. (2018b) with the primers TW81 and AB28 (Goff et al. 1994). For the strain NIES‐4476, the small and large nuclear subunits of rDNA (SSU and LSU rDNA, respectively), ITS2, ribulose‐1,5‐bisphosphate carboxylase/oxygenase large subunit gene (*rbc*L), chloroplast‐encoded ATP synthase beta subunit gene (*atp*B), and P700 chlorophyll a apoprotein A2 gene (*psa*B) regions were amplified and sequenced as described previously (Matsuzaki et al. 2019), using existing primers (Table S1 in the Supporting Information). The obtained sequences were deposited to the National Center for Biotechnology Information (NCBI) Nucleotide sequence database and available as ON065541 (ITS2 from the field sample WP187) and LC683781–LC683786 (SSU and LSU rDNA, ITS2, *rbc*L, *atp*B, and *psa*B of the strain NIES‐4476), respectively. Furthermore, longer *rbc*L fragments (1128 bp) of *Chloromonas nivalis* subsp. *tatrae* field cysts LP01 (Procházková et al. 2018b) and *Chloromonas hindakii* strain WP129/CCCryo 531‐19 (Procházková et al. 2019a) were acquired for phylogenetic analyses (using primers F1 and R3 in Table S1) and the acquired sequences were submitted to NCBI under accession numbers KY499615.2 and MN251877.2, respectively.

For molecular phylogeny of the strain NIES‐4476, Bayesian inference (BI) using MrBayes 3.2.7 (Ronquist et al. 2012) and maximum likelihood (ML) analysis using RAxML‐NG 0.9.0 (Kozlov et al. 2019) were conducted as described previously (Matsuzaki et al. 2019, 2021). The appropriate substitution models for each analysis were selected based on the Bayes Information Criterion, using ModelTest‐NG 0.1.6 (Darriba et al. 2020). Operational taxonomic units examined in each molecular phylogenetic analysis and their DDBJ/ENA/GenBank accession numbers are shown in Table S2 in the Supporting Information. All belong to *Chloromonadinia* clade (Nakada et al. 2008), and the outgroup was chosen based on previous phylogenetic results (Hoham et al. 2002, 2006). The methods of annotation and prediction of the secondary structure of ITS2 follow those described by Matsuzaki et al. (2014).

To search for sequences assignable to *Chloromonas kaweckae* in metagenomic data from previous studies, paired‐end amplicon data obtained from snow and glaciers using Illumina platforms were downloaded from the European Nucleotide Archive (https://www.ebi.ac.uk/ena/browser/home). Each Sequence Read Archive was converted to a fasta file using sratoolkit.2.11.2. In this dataset, *C. kaweckae* sequences were searched using Vsearch 2.15.1 (Rognes et al. 2016) with the following conditions: identity >0.9940 in 18S rRNA (i.e., maximally 2 bp nucleotide difference in e.g. a 342 bp sequence was allowed; Lutz et al. 2019) and >0.97 in ITS2, and query and reference coverage >95% (Matsuzaki et al. 2021). In several databases obtained by Illumina, only raw reads were available. Thus, the raw read FASTQ files were pre‐processed by “fastp” (Chen et al. 2018) and respective pair‐end reads (treated) were merged by “vsearch” and processed against the query file as mentioned above.

Cellular fatty acids of the field sample WP187 were extracted, derivatized, identified, and quantified by comparison of gas chromatography retention times and fragmentation patterns with those of calibration standard FAMEs (Supelco, Prague), using methods of Řezanka and Dembitsky (1999) and Vančura et al. (1988). Procedures were described in detail by Procházková et al. (2018b).

To measure the photosystem in the genuine state as it was found in situ, in vivo chlorophyll fluorescence parameters of the field sample WP187 were obtained with a pulse–amplitude modulated fluorometer (PAM 2500, HeinzWalz GmbH, Germany). Field‐collected cells were put in a 0.6 mL chamber and cooled with an ice bath to approximately 2°C. Prior to measurement, algae were kept in the snow meltwater in the dark for 30 min. Then, to obtain the relative electron transport rate (rETR) and the light saturation point I_k_, cells were exposed to photon flux densities (PFD) of 5, 9, 34, 67, 104, 201, 366, 622, 984, 1389, 1666, and 2018 μmol photons · m^−2^ · s^−1^ for 30 s each. Four independent replicates were carried out; each replicate was one fresh biological sample (subsample of WP187), only once used for the P‐I curve. For further details, see Procházková et al. (2018b).

## 3 RESULTS

### 3.1 Taxonomic description

#### 3.1.1 C. kaweckae Procházková and Matsuzaki sp. nov. Figure 1

**Fig. 1:**
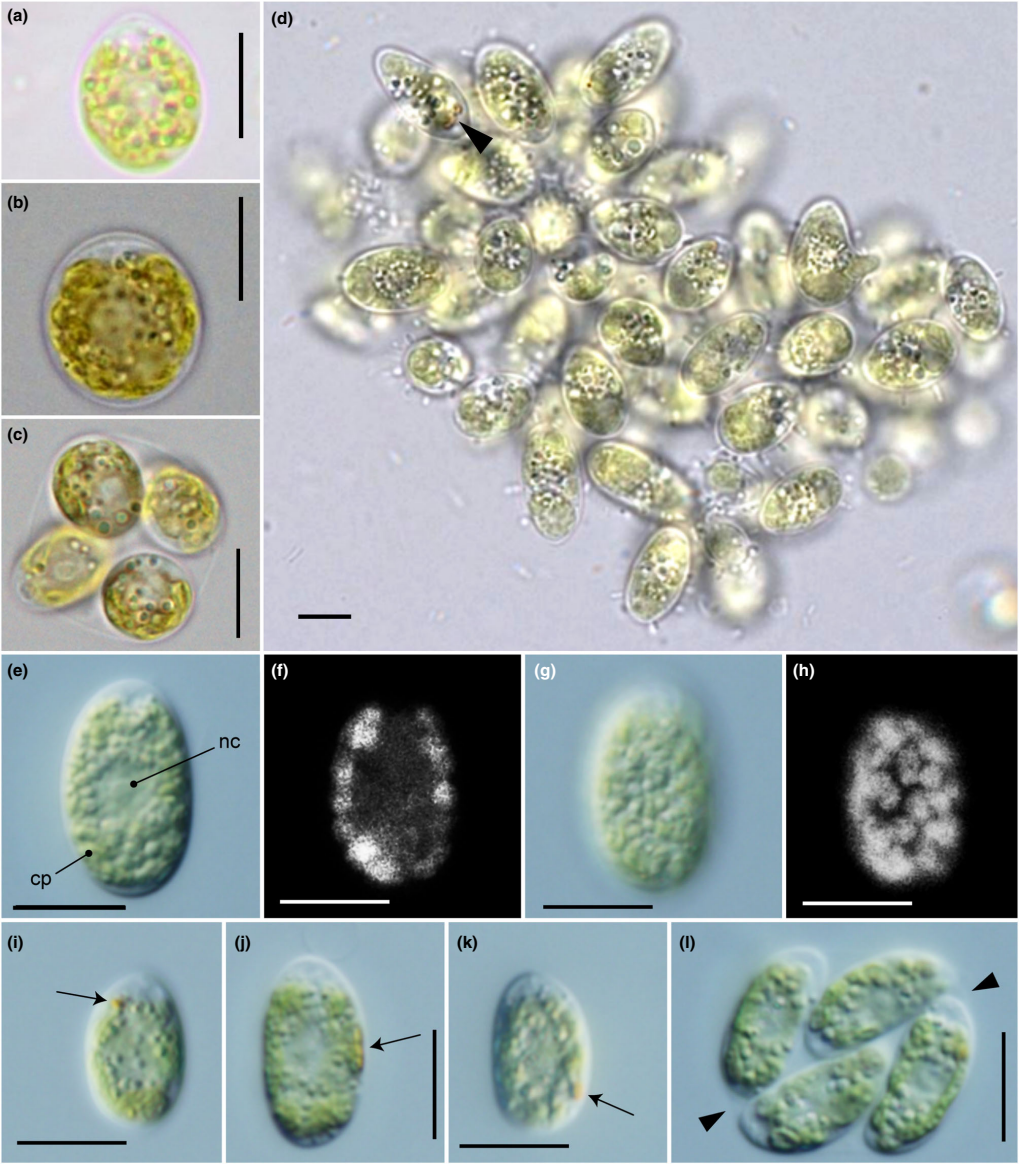
Light micrographs of *Chloromonas kaweckae*. (a–d) Field sample WP187. Scale bars = 10 μm. (a, b) Swarmer‐like stages, kept in snow meltwater (a) or at 1°C in BBM medium (b); however, without flagella anymore. Each median view shows cytoplasm with a central nucleus and a very perforated chloroplast. (c) Sporangium was kept at 1°C in BBM medium, consisting of four daughter cells. (d) Elongate cells with the two stigmas (arrow), apparently arranged in loose clusters, which appeared after keeping at 5°C for 2 days after harvest of a field sample of WP187. (e–l) Strain NIES‐4476. Identical magnification throughout (scale bars = 10 μm). (e–k) Vegetative cells. The anterior end of the cell is arranged upward. (e) Optical section. cp, chloroplast; nc, nucleus. (f) Epifluorescence image of (e). (g) Surface view. (h) Epifluorescence image of (g) showing the chloroplast seemingly composed of angular discs. (i) Eyespot positioned in the anterior third of the cell (arrow). (j) Eyespot positioned near the equator of the cell (arrow). (k) Eyespot positioned in the posterior third of the cell (arrow). (l) Autosporangium with four daughter cells within the parental cell wall. Arrowheads, parental cell wall. [Color figure can be viewed at wileyonlinelibrary.com]

Description: Vegetative cell solitary, with two flagella, without prominent anterior papilla. Cells broadly ovoid, 7.4–12.8 μm wide and 9.8–16.7 μm long in the field sample and ovoid, elongate‐ovoid to ellipsoidal, 9.2–15.5 μm wide and 16.0–22.5 μm long in culture. Cells with a central nucleus and single cup‐shaped chloroplast. Chloroplast is seemingly composed of angular disks, with irregularly incised surfaces and eyespot, without pyrenoids. Eyespot ellipsoidal, variably positioned in the posterior third to the anterior third of the cell. Asexual reproduction by the formation of usually two or four zoospores; protoplast rotation prior to the first cell division. Cell aggregates not observed in culture. Sexual reproduction is unknown. Possible motile zygotes observed in 2‐day‐incubated field samples elongate, 7.1–10 μm wide and 13.1–22 μm long, with two parallel stigmas. Zygotes or cysts morphology is unknown.

holotype (designated here): Specimen TNS‐AL‐58992 deposited at TNS (National Museum of Nature and Science, Tsukuba, Japan); material consists of resin‐embedded vegetative cells from strain NIES‐4476 (metabolically inactive state).

Strain examined: NIES‐4476 (= CCCryo 557‐22).

Type locality: Green snow close to Veľké Spišské pleso, Malá Studená dolina, Vysoké Tatry, Slovakia; N 49°11.515, E 20°11.628, altitude 2026 m a.s.l.

Etymology: The species epithet “*kaweckae*” is in honor of the phycologist Prof. Barbara Kawecka, Polish Academy of Sciences, who has significantly contributed to taxonomy, ecology, and biology of snow‐dwelling microalgae (e.g., Starmach and Kawecka 1965, Kawecka and Drake 1978, Kawecka et al. 1979, Kawecka 1981, Kawecka 1983/84, Kawecka and Eloranta 1986).

#### 3.1.2 Habitat description

**Fig. 2:**
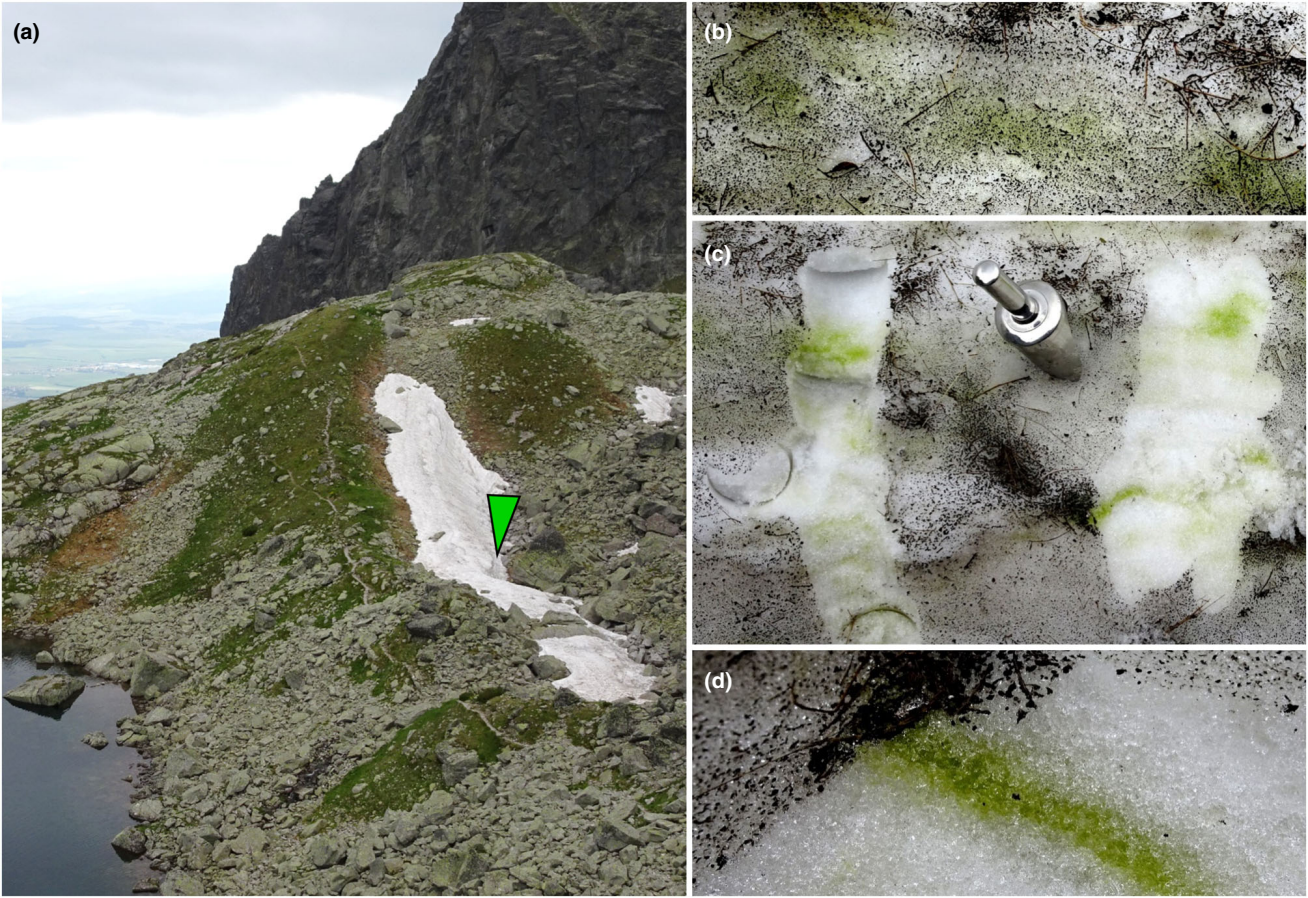
Overview of the sampling site in the upper part of Malá Studená Valley, High Tatra Mountains (Slovakia) with a green bloom caused by *Chloromonas kaweckae*. The snow surface was moreover covered by blackish detritus. Photos were taken on 16 June 2018: (a) The snowfield was bedded on a modest slope in a field depression (arrow: bloom). To the left the shore of the lake Veľké Spišské pleso. (b) Green spots were visible on the snow surface, here still covered by dark detritus before removing with a scoop. (c) Superficial “black snow” (likely airborne particles) was abundant. (d) The highest algal cell density was from the surface down to 3 cm below the snow surface. [Color figure can be viewed at wileyonlinelibrary.com]

The alpine sampling site was located on a modest slope in a south‐west orientated field depression (Fig. 2). The snowfield was situated above timberline, covering a combination of alpine meadow and granitoid boulders (Fig. 2a). The snow surface was blackish green (Fig. 2b), starting to be pure green approximately 0.5 cm below, and the bloom reached down to 3 cm depth (Fig. 2c). The dark aspect (“black debris”) was formed by nonbiological particles which did not reach into deeper layers (Fig. 2d). In the field microscope, the sample was dominated by *Chloromonas‐*like cells. The meltwater had an almost neutral pH of 6.8 and an electrical conductivity of 3.3 μS · cm^−1^.

#### 3.1.3 Maximal population density and morphology of field cells

Light microscopy of the field sample WP187 indicated that the green snow color was caused by a virtually monospecific bloom of a chlamydomonadacean alga with a distinctively perforated, pyrenoid‐free chloroplast. A minor component with less than 1% of cells was thick‐walled cysts of *Chloromonas* cf. *nivalis*. The main constituents of the bloom were swarmer‐like, broadly ovoid cells, however, lacking flagella (Fig. 1a). No cell division stages of the alga were observed in the snow with field microscope (and confirmed by LM observation of Lugol fixed field sample). Cell size ranges were 7.4–12.8 μm width and 9.8–16.7 μm length (*n* = 50, Lugol fixed field sample WP187). The maximal population density was 9.34 × 10^6^ ± 4.67 × 10^5^ green cells · mL^−1^ meltwater.

After 2 d of incubation of the field sample WP187 at 5°C and low illumination, LM revealed the presence of the same life cycle stages (Fig. 1b) as those observed in the field as well as further but less abundant ones, such as copulating spherical cells, putative planozygotes with four flagella and sporangia with two or four daughter cells (Fig. 1c). Interestingly, prominent elongate stages with two parallel stigmas (likely in its transition to the cyst stage; 7.1–10 μm wide and 13.1–22 μm long; *n* = 30; Fig. 1d) became dominant over the initially harvested, subspherical stages. Further life cycle stages were not observed.

#### 3.1.4 Morphological characterization of *Chloromonas kaweckae* sp. nov. strain NIES‐4476

Under light and fluorescent microscopy of lab strain NIES‐4476 originating from the field sample WP187 (Fig. 1, e–l), vegetative cells were ovoid, elongate‐ovoid to ellipsoidal. The cells were 9.2–15.5 μm wide and 16.0–22.5 μm long. Each cell had two equal flagella at the anterior end, a single chloroplast, two contractile vacuoles near the base of the flagella, and a single nucleus, without a prominent anterior papilla (Fig. 1e). The chloroplast was cup‐shaped with an eyespot and lacked pyrenoids (Fig. 1, e–l). The surface view of the chloroplast had irregular incisions, seemingly composed of angular discs (Fig. 1, g and h). The eyespot was ellipsoidal, variably positioned in the anterior third to the posterior third of the cell (Fig. 1, i–k). The nucleus was almost spherical, located in the center of the protoplast (Fig. 1e). The flagella were of equal length to the whole cell. Asexual reproduction was via zoospore formation. As described in other snow *Chloromonas* species (e.g., Matsuzaki et al. 2014, 2018), the protoplast rotated just before the first cell division. Generally, two or four daughter cells were produced within the parental cell wall (Fig. 1l). Aggregates of 16 or more cells resulting in the repeated division of daughter cells within the parental cell wall (e.g., Matsuzaki et al. 2018) were not produced even in 2‐month‐old cultures. Sexual reproduction and production of cysts or zygotes were not observed in the culture, even when using the induction method for *Chloromonas fukushimae* and *C. tughillensis* (Matsuzaki et al. 2020). Cells of *C*. *kaweckae* showed no growth at 15°C after cultivation for 2 weeks, as described in previous reports of other snow‐inhabiting *Chloromonas* species (e.g., Matsuzaki et al. 2019), this may suggest that it represents a psychrophile (i.e., cold‐loving organism with growth temperature optima below 15°C).

#### 3.1.5 Molecular phylogenetic analyses

**Fig. 3:**
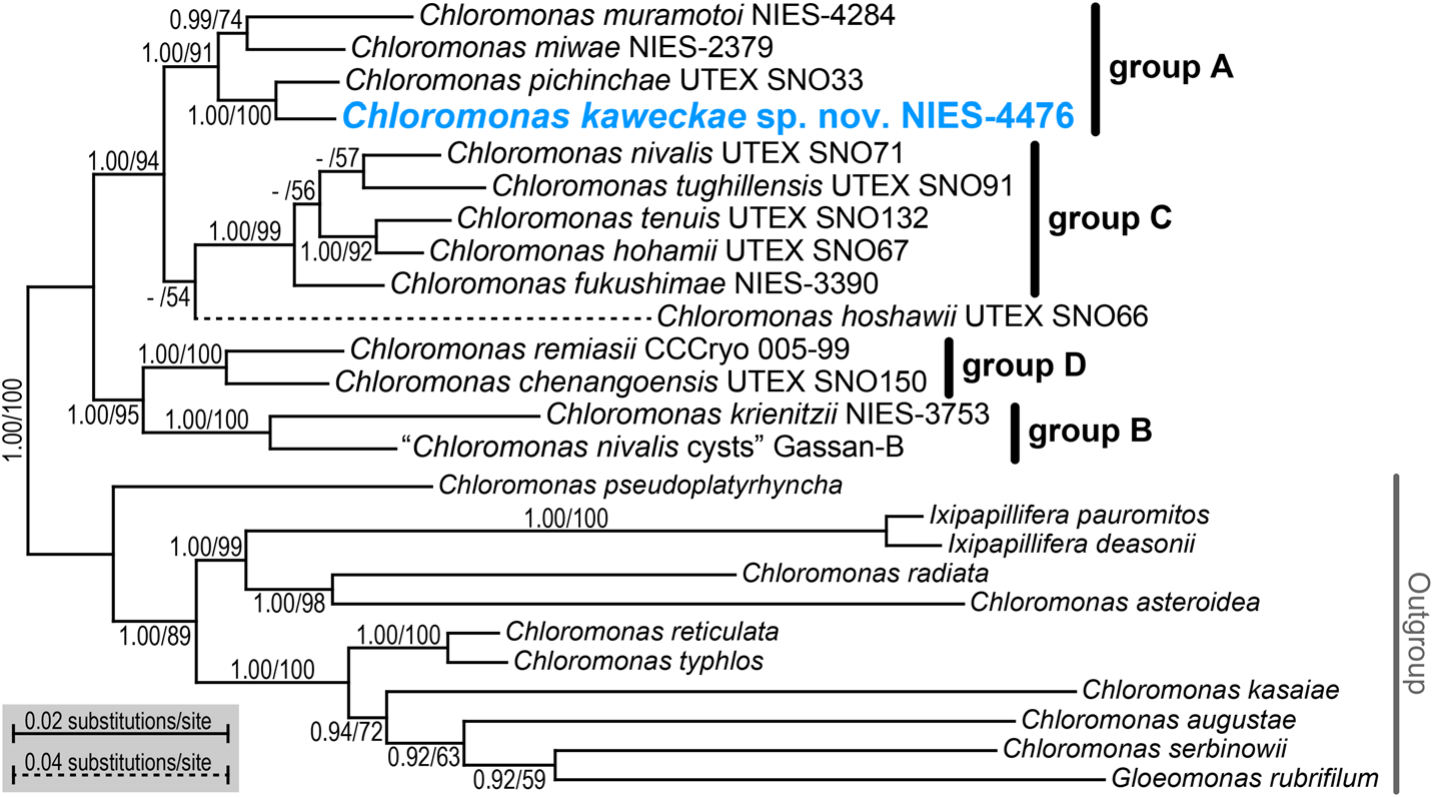
Bayesian phylogenetic tree based on 5383 bp of the concatenated nuclear SSU and LSU ribosomal DNA genes and first and second codon positions of *atp*B and *psa*B. The applied substitution models are K80 + I + G4 for SSU rDNA, GTR + I + G4 for LSU rDNA and first codon position of *atp*B and *psa*B, and F81 + I + G4 for 2nd codon position of *atp*B and *psa*B. Clade names are indicated according to Matsuzaki et al. (2019). The corresponding posterior probabilities of Bayesian inference (0.90 or more, left) and bootstrap values from a maximum likelihood analysis (50% or more, right) are shown at each node. [Color figure can be viewed at wileyonlinelibrary.com]

The phylogenies of SSU rDNA (Fig. S1 in the Supporting Information) and *rbc*L markers (Fig. S2 inthe Supporting Information) comprising *Chloromonas* species living in the snow indicated that the new strain NIES‐4476 is a member of a clade named “group A" by Matsuzaki et al. (2019). This clade further consisted of three species examined previously (*Chloromonas pichinchae* UTEX SNO33, *C. muramotoi* NIES‐4284, and *C. miwae* NIES‐2379; Muramoto et al. 2010, Matsuzaki et al. 2014, 2019) and strains preliminarily assigned to *C.* cf. *rostafinskii* (CCCryo 010‐99 and CCCryo 025‐99) and *Chloromonas* cf. *alpina* CCCryo 032‐99. However, there was no robust statistical support for the monophyly of the clade, and phylogenetic relationships between the strain NIES‐4476 and other snow *Chloromonas* spp. were not well resolved in either analysis. On the contrary, the monophyly of group A was recovered with strong statistical supports (1.00 posterior probabilities [PP] in BI and 91% bootstrap values [BV] in ML) in the phylogenetic analysis based on SSU and LSU rDNA and *atp*B and *psa*B (Fig. 3); the regions were used for the previous molecular phylogenies of snow *Chloromonas* (Matsuzaki et al. 2018, 2019). As indicated by SSU‐ and *rbc*L‐based phylogenetic trees (Figs. S1 and S2), the strain NIES‐4476 was included in the clade “group A" together with *C. miwae*, *C. muramotoi*, and *C. pichinchae*. Within the clade, strain NIES‐4476 was sister to *C. pichinchae* (Fig. 3) with 1.00 PP in BI and 100% BV in ML analyses.

#### 3.1.6 Comparison of genetic differences between the strain NIES‐4476 and *Chloromonaspichinchae*

**Fig. 4:**
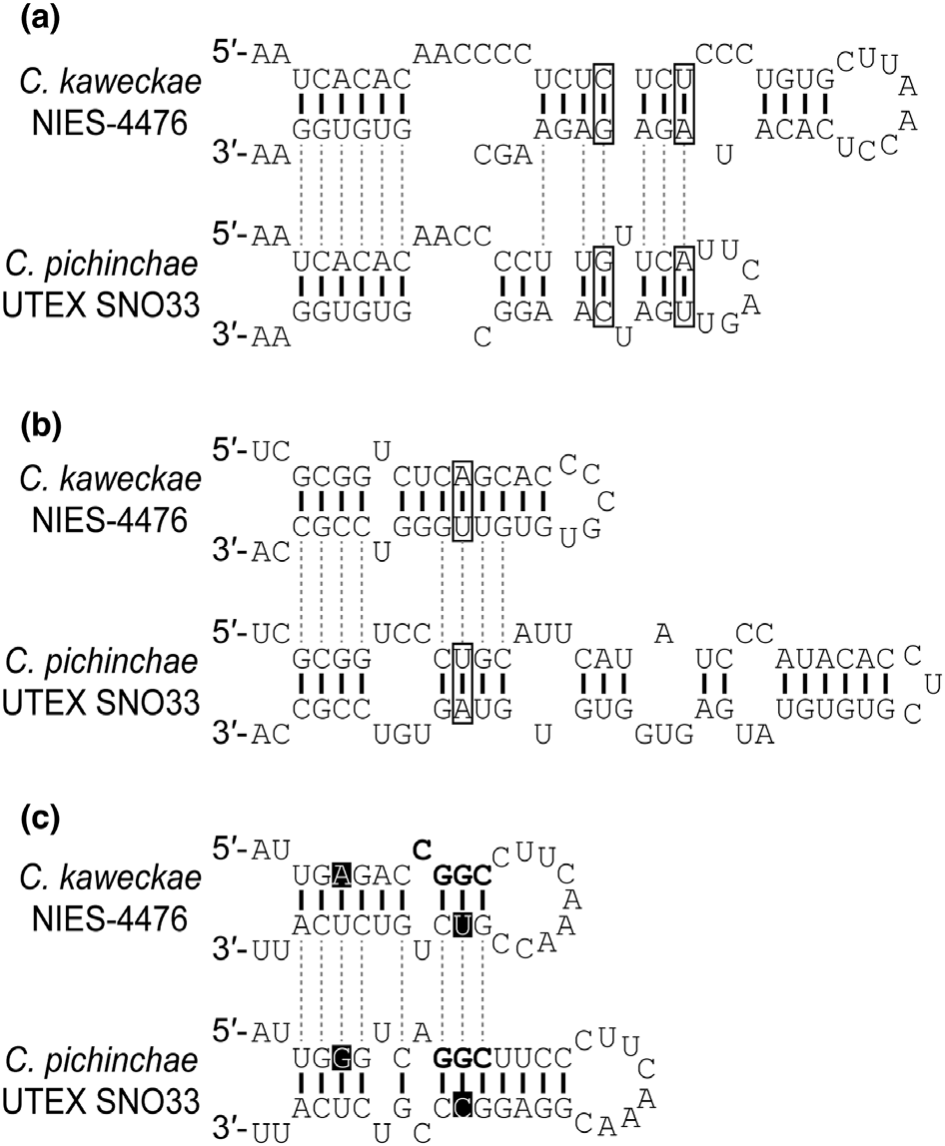
Comparison of the ITS2 secondary structures between *Chloromonas kaweckae* NIES‐4476 and *C. pichinchae* UTEX SNO33. Compensatory base changes and hemi‐compensatory base changes are indicated by open boxes and black backgrounds, respectively. (a) Helix I. (b) Helix II. (c) Apex near the YGGY motif (boldfaces) in helix III, the most conserved region of ITS2. For the complete secondary structure, see Figure S3.

The partial ITS2 sequence from the field material (WP187) turned out to be identical to that of the strain NIES‐4476 (Fig. S3 in the Supporting Information), which showed that this strain originates from the massive bloom (Fig. 2). To confirm whether strain NIES‐4476 is a distinct species from the closest known relative, *C. pichinchae* or otherwise, we compared the secondary structures of their ITS2 regions (Fig. S3). As shown in Figure 4, structural differences correlating with the separation of biological species were observed in the helices I and II, and some branches in helix III between the strain NIES‐4476 and *C. pichinchae*. In detail, two and one compensatory base changes (CBCs) were detected in helix I and helix II, respectively (Fig. 4a and b). Furthermore, two hemi‐CBCs were found at the apex near the branch encompassing the YGGY motif of ITS2 helix III, the most conserved region of ITS2 (Coleman 2003; Schultz et al. 2005; Fig. 4c).

**Fig. 5:**
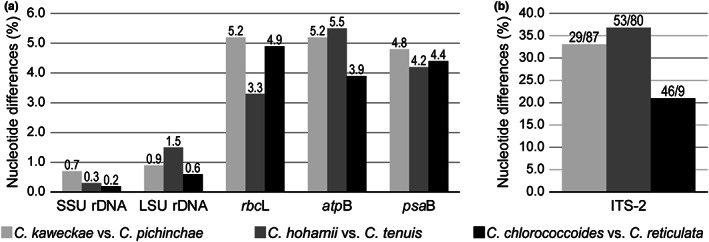
Genetic differences between three pairs of closely related *Chloromonas* species. Gray: *C. kaweckae* NIES‐4476 and *C. pichinchae* UTEX SNO33. The nucleotide differences between the sister species, *C. hohamii* UTEX SNO67 and *C. tenuis* UTEX SNO132 (dark gray), and *C. chlorococcoides* SAG 15.82 and *C. reticulata* SAG 29.83 (black), are according to the previous study (Matsuzaki et al. 2014) and this study. (a) Uncorrected p‐distances (%) from pairwise comparisons in five genes: 1746 bp of SSU rDNA; 2013 bp of LSU rDNA; 1128 bp of *rbc*L; 1128 bp of *atp*B; and 1392 bp of *psa*B. (b) Uncorrected p‐distances (%) from pairwise comparisons in ITS2. The number of nucleotide differences (left) and gaps (right) are shown on each bar.

In addition, we compared nucleotide differences (uncorrected p‐distances) in the five molecular markers used in the present phylogenetic analyses (nuclear‐encoded SSU and LSU rDNA, and plastid‐encoded *rbc*L, *atp*B and *psa*B) and ITS2 between strain NIES‐4476 and *C. pichinchae* (Fig. 5, a and b). Nucleotide differences in each of these six regions between the two were almost identical to those between other pairs of sister species within *Chloromonas* (*C. hohamii* and *C. tenuis*; and *C. chlorococcoides* and *C. reticulata*, respectively), where separation was studied based on both morphological and molecular data (Matsuzaki et al. 2012, 2014).

### 3.2 Fatty acid composition

**Fig. 6:**
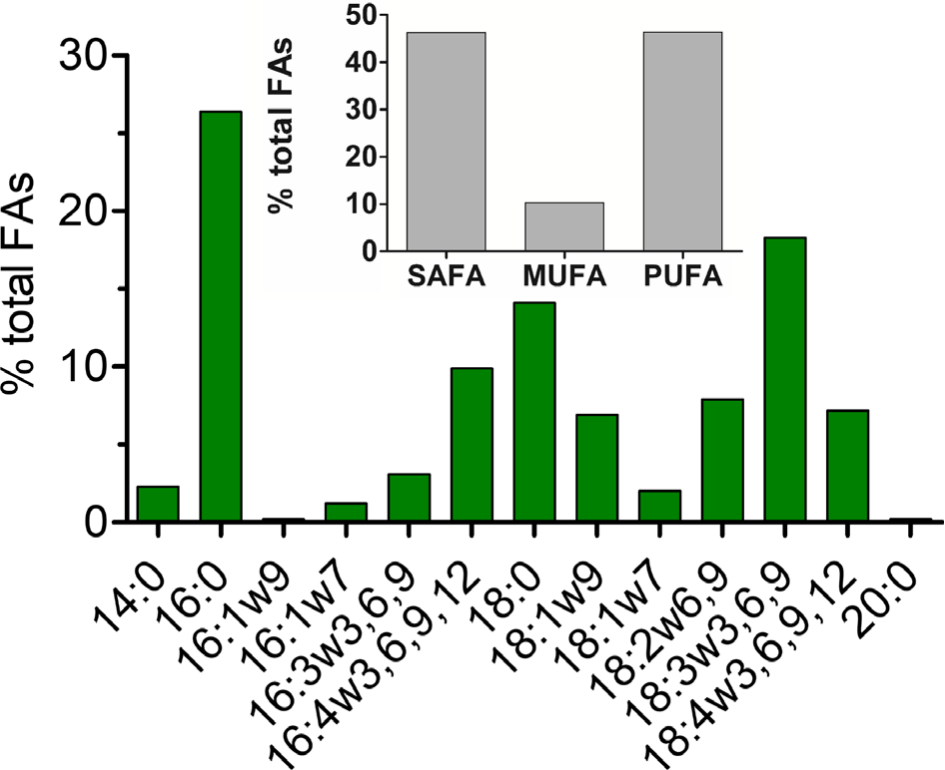
Cellular fatty acid composition in swarmer‐like stages of *Chloromonas kaweckae* (field sample WP187) in [%] of total fatty acids. The relative proportion of saturated (SAFA), monounsaturated (MUFA), and polyunsaturated (PUFA) fatty acids is given in the inset. The figure shows only fatty acids that had abundances greater than 0.1% of total fatty acids. [Color figure can be viewed at wileyonlinelibrary.com]

The relative cellular content of FAs (in the percentage of total fatty acids) in *C. kaweckae* field vegetative stages (WP187) is shown in Figure 6. FAs of chain length C14 to C20 were found. The cells showed elevated proportions of polyunsaturated fatty acids (PUFAs) (46.4% of total fatty acids) and the content of saturated acids (SAFAs) reached 43.3% (mainly palmitic acid, 16:0, 26.4%). The main monounsaturated fatty acid (MUFA) was oleic acid (18:1 (9Z), 6.9%). The dominant PUFA was linolenic acid (18:3 [9Z, 12Z, 15Z], 18.3%), followed by hexadecatetraenoic acid (16:4 [4Z, 7Z, 11Z, 13Z], 9.9%).

### 3.3 Photosynthesis

**Fig. 7:**
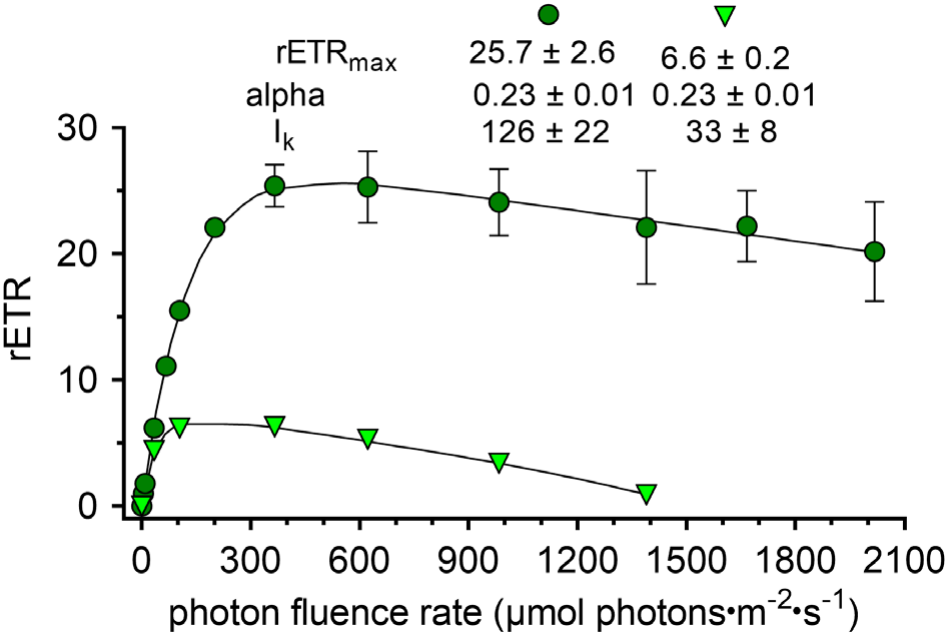
Effect of increasing photon fluence rates (*x*‐axis) on the relative electron transport rate (rETR; *y*‐axis) of chloroplasts in swarmer‐like stages of *Chloromonas kaweckae* (circles, field sample WP187, this study) and *Chloromonas hindakii* (triangles, strain WP129/CCCryo 531‐19, Procházková et al. 2019a). Values are means of four replicate measurements. The data points were fitted to the photoinhibition model of Walsby (1997). [Color figure can be viewed at wileyonlinelibrary.com]

Light‐dependent relative electron transport rates revealed the extent of adaptation of PSII to the habitat conditions, measured at ambient temperature. The field cells of *Chloromonas kaweckae* WP187 were photophysiologically active and not dormant. They exhibited an α (slope in the light‐limited section of the photosynthesis‐irradiance curve; P‐I) of 0.23 ± 0.01, a relative ETR_max_ of 25.7 ± 2.6, and an I_k_ value of 126 ± 22 μmol photons · m^−2^ · s^−1^ (Fig. 7). Signs of photoinhibition started at irradiances greater than 600 μmol photons · m^−2^ · s^−1^ (as it is indicated by the decreasing slope of the P‐I line in Fig. 7). For comparison, data from another *Chloromonas* species from the snow above timberline was included in Figure 7 (Procházková et al. 2019a). This showed that I_k_ parameter was almost four times lower for *C. hindakii* WP129/CCCryo 531–19 and photoinhibition occurred already above 200 μmol photons · m^−2^ · s^−1^, indicating that high irradiance does not favor vegetative stages.

## 4 DISCUSSION

The observed black debris (inorganic particles) in surface snow layers (Fig. 2, b–d) seems to be of airborne origin, and it seems to be typical for the High Tatras during summer (e.g., Kol 1966, Procházková et al. 2020). It can reduce snow surface albedo and shading may support favorable conditions for the observed green bloom by reducing excessive irradiation. The slightly acidic pH and very low electrical conductivities of meltwater were in accordance with what was reported earlier in the region (Procházková et al. 2019a). Generally, transient green stages of *Chloromonas* spp. in snow can be easily overlooked in sub‐alpine and alpine habitats of lower latitudes, because blooms are either small, or more significantly, the green cells are usually “hidden” below a white snowpack in the early melting period. Nonetheless, green surface blooms caused by *Raphidonema*‐like algae (Trebouxiophyceae) have been occasionally reported from the Alps from exposed sites (Kol 1961), while reports of these algae in the High Tatra Mts. were from very shaded locations at lower altitudes (Komárek et al. 1973). *Carteria győrffyi* (Kol 1949) and *C. kaweckae* sp. nov. (this study) are unique examples of a chloromonad causing surface green snow at light‐exposed sites as vegetative stages since such blooms have rarely been reported in nonpolar regions. In contrast, green surface snow seems to be common at forested sites (Nedbalová et al. 2008, Ono et al. 2021). For instance, the vegetative cells of *C. pichinchae* were found in heavily shaded spots near the bases of coniferous trees, in diffuse green patches near the surface, and in distinct green horizontal bands at depths from 2 to 24 cm below the surface of snowbanks (Hoham 1975). Using protocols described by Gates (1980), the measurement of Hoham (1975; the effective band 440–910 nm; at 12:30–12:45 hours) of 46.46 mW · cm^−2^ can be converted to quantum units. Incident midday photosynthetically active radiation (PAR) levels for *C. pichinchae* during fine weather at Washington Stuart Range, 1387 m a.s.l., USA (Hoham 1975) was 1965 μmol photons · m^−2^ · s^−1^, being lower in comparison with high‐alpine sites in the High Tatras (up to 2500 μmol photons · m^−2^ · s^−1^; Procházková et al. 2018a). Given the fact that light penetration decreases logarithmically with increasing snow depth (Gorton et al. 2001), algal cells found 2 cm below snow surfaces experienced about 30% of total irradiance (i.e., maximally ~600 μmol photons · m^−2^ · s^−1^ for *C. pichinchae* during noon under the clear sky; Hoham 1975). Therefore, maximum light conditions for vegetative stages of *C. pichinchae* were approximately four times lower when compared to those midday incident readings at the snow surface for *C. kaweckae* in the High Tatras. However, during the field campaign of this study, no division cell stages were observed in snow, so exact light conditions for the growth of this species are unknown. It is not excluded that cell divisions had happened earlier during foggy days under dim light conditions or when cells were located deeper in the snowpack. Interestingly, *Chlainomonas koliae* at Mt Philistine, New Zealand, was reported to survive periods of high irradiances, but in contrast was also found to undertake growth during lower light for almost 1 week of continuous clouds with rain (PAR 170 μmol photons · m^−2^ · s^−1^), or during storm events (Novis 2002). We cannot exclude that *C. kaweckae* forms protective cyst stages containing shielding secondary carotenoids later during the melting season, but usually *Chloromonas* cells start this transformation process before reaching the snow surface.

Although we found green surface snow caused by chloromonads also at other sites of the High Tatras (data not shown), it remains unknown how geographically widespread blooms of *C. kaweckae* are. Generally, scattered snow algal cells can be found in visually white snow (Hanzelová et al. 2018) or in lake water during and after the snowmelt (Lukavský 1994, Novis 2002, Nedbalová et al. 2006, Lenarczyk and Tsarenko 2013). From a biogeographic perspective, microbial snow communities either harbor cosmopolitan taxa (Procházková et al. 2019b), widely distributed ones (Procházková et al. 2018a, 2020), or only regionally reported ones (Procházková et al. 2018b). Sequences of 18S rDNA assignable to *C. kaweckae* (or at least to close relatives) were found elsewhere in low numbers (<1% total reads) in meta‐amplicon sequencing data (Table S3 in the Supporting Information) of snow in British Columbia (Yakimovich et al. 2020), in red snow at Mt Asahi in Japan (Terashima et al. 2017), in red, slightly black, brown, or orange snow in Mt. Tateyama in Japan (Nakashima et al. 2021; T. Nakashima, pers. comm.) and red snow in Austrian Alps (Lutz et al. 2019). This indicates that *C. kaweckae* was not endemic to the sampling site of this study. While scattered cells of microalgae can have a wide geographic distribution, it remains unknown why *C. kaweckae* apparently does not dominate cryoflora communities frequently, and furthermore which environmental conditions select for a bloom of this particular species.

The presented phylogenetic analyses (Figs. 3, S1, and S2) showed that *Chloromonas kaweckae* belongs to the *Chloromonadinia* clade (Nakada et al. 2008) which corresponds to the genus *Chloromonas* sensu Pröschold et al. (2001). Within the clade, *C. kaweckae* was positioned into a monophyletic group composed entirely of snow‐inhabiting species (subclade 2 of clade A, Hoham et al. 2002, 2006 or SA clade, Matsuzaki et al. 2013) and was closely related to *C. pichinchae* UTEX 33 of North American origin (Fig. 3). Between *C. kaweckae* and *C. pichinchae*, we found at least three CBCs in the helices I and II and two hemi‐CBCs near the YGGY motif in the helix III in ITS2 secondary structures (Fig. 4). In the case where a CBC among the entire helices of ITS2 is present, two specimens can be regarded as different with a probability of ~0.93 (Müller et al. 2007, Wolf et al. 2013). In addition, Coleman (2009) reported that organisms with even one hemi‐CBC near the YGGY motif show a weak degree of interbreeding. Moreover, the nucleotide differences in the six DNA regions between *C. kaweckae* and *C. pichinchae* are almost identical to those between two other sister species of *Chloromonas* of which the separation was examined previously (Fig. 5). Therefore, these genetic distances indicated that *C. kaweckae* is distinct from *C. pichinchae*. Furthermore, *C. kaweckae* also differs from *C. pichinchae* in ecology since the latter was reported from snow in heavily shaded areas near the bases of coniferous trees (Hoham 1975).

Based on light microscopy, the vegetative cell of *C. kaweckae* in culture resembled those of 10 other snow‐inhabiting species of *Chloromonas* (*C. alpina*, *C. bolyaiana*, *C. brevispina*, *C. chenangoensis*, *C. hohamii*, *C. miwae*, *C. muramotoi*, *C. pichinchae*, *C. remiasii*, and *C. tughillensis*) in having ovoid, elongate‐ovoid, or ellipsoidal cell shape without prominent anterior papilla (Table S4 in the Supporting Information). However, *C. kaweckae* is distinguishable from these 10 species by cell size, chloroplast morphology, eyespot position and shape, up to 4 zoospores within the parental cell wall during asexual reproduction, and the absence of production of cell aggregates in old cultures (Table S4; Appendix S1 in the Supporting Information). For *Chloromonas* species not described from melting snow, *C. gutenbrunnensis* and *C. hyperstigmata* are most similar to *C. kaweckae*, in possessing ovoid vegetative cells and a cup‐shaped chloroplast with irregular incisions and in lacking a prominent anterior papilla. However, cell size, eyespot position, and eyespot characteristics are different: *C. gutenbrunnensis* reaches up to 23 μm width and up to 27 μm length (Wawrik 1974; vs. 9.2–15.5 μm wide × 16–22.5 μm in *C. kaweckae*), and the eyespot is located in posterior half only (Wawrik 1974; vs. posterior third to anterior third of the cell in *C. kaweckae*). *C. hyperstigmata* possesses quite a large disc‐shaped eyespot (diameter up to one‐fourth of the cell length; Ettl 1961), whereas such a large eyespot has never been observed in *C. kaweckae*. As a consequence, *C. kaweckae* represents morphologically and genetically an independent, new species of the genus *Chloromonas*.

The whole life cycle of *Chloromonas kaweckae*, such as cysts or zygote morphology and sexual reproduction, remains unknown. In general, cryoflora species of *Chloromonas* typically form striking cysts or zygotes with rigid cell walls, being the dominant stages in time. *C. pichinchae*, *C. miwae*, and *C. muramotoi* (which form a robust clade together with *C. kaweckae*; Fig. 3) were found to make robust “*Scotiella*”‐like cysts or zygotes with raised surface ridges (Hoham 1975, Matsuzaki et al. 2015, 2019). Such cells accumulate secondary carotenoids in cytosolic lipid bodies, causing orange‐reddish snow blooms.

The phenomenon of massive green surface snow is related to polar coastal regions which receive a lot of rain/snow/wetness due to prevailing winds from Ocean (T. Leya, pers. comm.; Bidigare et al. 1993, Leya 2013, Soto et al. 2020, Gray et al. 2021, Khan et al. 2021). There, lower PAR prevails (Ambach et al. 1991), and due to animal colonies more nutrients useful for repair mechanisms are available than at alpine locations (coastal influenced by marine life, e.g., Gray et al. 2020).

The analysis of field sample WP187 showed that the population of *Chloromonas kaweckae* was rich in unsaturated fatty acids. In addition, α‐linolenic acid (18:3n3) was the dominant unsaturated cellular FA, which is consistent with profiles of other *Chloromonas* species from snow or of strains kept in nitrogen‐deficient medium (Spijkerman et al. 2012; Fig. 6). This reflects the major role of PUFAs avoiding the rigidity of cell membranes in cold environments (Morgan‐Kiss et al. 2006).

The fluorometric measurements (Fig. 7) demonstrated that the PSII of *Chloromonas kaweckae* was adapted to relatively high irradiance levels in situ. Based on these short‐term responses, signs of photoinhibition were noticed from approximately 600 μmol photons · m^−2^ · s^−1^ on. Long‐term adaptations like PS re‐arrangement or shielding by secondary carotenoid accumulation (the latter in the cyst stage only) may also take place, but this was not covered by the current study. Given the fact that most cells stayed in the uppermost 3 cm of the snowpack, and considering the black surficial detritus causing partial shading, likely not all the population suffered from excessive PAR. Still, the extent of high‐light adaptation was remarkable for *Chloromonas* green stages, because significant photon fluence rates were still recorded at higher irradiance levels (<2100 μmol photons · m^−2^ · s^−1^). Irradiance conditions during sampling were not possible to be measured, but it was a sunny day and prevailing light conditions are known from a nearby mountain valley at a similar elevation where PAR at full sunlight was up to 2500 μmol photons · m^−2^ · s^−1^ (Procházková et al. 2018a). PSII of *C. kaweckae* was adapted to three times higher light conditions compared to flagellates of *C. hindakii* (Procházková et al. 2019a), another snow alga recently characterized in the same mountain range, and even more compared to cyst stages of *Scotiella cryophila* from deep subsurface green snow in the Alps (Remias et al. 2018).

But how can the green microalgae actually cope with high irradiance? In a presumed oligotrophic habitat like alpine melting snow, PSII repair, namely an increased rate of turnover of photodamaged proteins in PSII, seems to be limited (Bidigare et al. 1993). Consequently, an alternative strategy, like enhancing the cyclic electron transfer around photosystem I can reduce the light‐harvesting ability of PSII (Dolhi et al. 2013). Alternatively, vacuoles with crystalline content rich in nitrogen (e.g., in form of guanin) may represent a so far underestimated intracellular source. They were morphologically observed in snow‐dwelling *Chloromonas* vegetative cells (Procházková et al. 2019a) and could serve for PS turnover reserves or repair mechanisms (Mojzeš et al. 2020). Eventually, the new strain of *C. kaweckae* will facilitate elucidating photosynthetic high light adaptations in terms of nonphotochemical quenching (Allorent et al. 2013).

Snow algae above timberline are exposed not only to high PAR but also elevated UV‐A and UV‐B. The contribution of UV to total solar radiation increases with altitude because of a thinner, relatively unpolluted, and more transparent atmosphere in many mountainous regions (Blumthaler et al. 1997). Interestingly, UV‐B was reported to induce photoprotective regulation of photosynthetic activity in the chloroplast in *Chlamydomonas reinhardtii* (Allorent et al. 2016). Although beyond the scope of this study, the ability of *C. kaweckae* to handle high light and remain green in surface snow could be due to a combination of some of these mechanisms, substituting the lack of shading astaxanthin, at least in the stage how they were found during harvest. Additionally, *C. kaweckae* was also able to perform photosynthesis well under low light conditions: a high alpha value of 0.23 supports the performance of cells located deeper below the snow surface.

In conclusion, extremophilic habitats like melting snow harbor diverse cryoflora communities, and the new species *Chloromonas kaweckae* is the first chloromonad to be characterized by an alpine green snow bloom. Cellular fatty acid profiles and high‐light adapted photophysiological characteristics indicate that these green vegetative stages were able to cope with the harsh conditions in surface layers of melting snowpack above timberline, which is in contrast to the majority of snow *Chloromonas* species.

## Supporting information


**Appendix S1.** Key to vegetative cells of snow‐inhabiting species of *Chloromonas* sensu Ettl (1970, 1983). The key is mainly based on the previous key (Matsuzaki et al. 2018). Species examined using cultured material are marked with asterisks (on the basis of the supplemental references and the present study).

**Figure S1.** Bayesian phylogenetic tree of snow inhabiting *Chloromonas* spp. based on 1,621 bp of nuclear encoded SSU ribosomal DNA (18S rDNA gene). The applied substitution model was K80 + I + G4. Clade names are indicated according to Matsuzaki et al. (2019). The corresponding posterior probabilities of Bayesian inference (0.90 or more, left) and bootstrap values from a maximum likelihood analysis (50% or more, right) are shown at each node (Matsuzaki et al. 2019).

**Figure S2.** Bayesian phylogenetic tree based on 1,128 bp of the *rbc*L gene. The applied substitution models are GTR + I + G4 for the first and third codon positions and JC + I for the second codon position. Clade names are indicated according to Matsuzaki et al. (2019). The corresponding posterior probabilities of Bayesian inference (0.90 or more, left) and bootstrap values from a maximum likelihood analysis (50% or more, right) are shown at each node.

**Figure S3.** Secondary structure of ITS2 transcript of *Chloromonas kaweckae* NIES‐4476 (accession number: LC683783). The 3′ end of the 5.8 S rRNA and the 5′ end of the LSU rRNA are shown. Differences between the strain and *Chloromonas pichinchae* strain UTEX SNO33 (accession number: LC012761) are described just outside the structure as blue characters. Note U–U mismatch in helix II (arrowheads) and the YGGY motif on the 5′side near the apex of helix III (boldface), common structural hallmarks of eukaryotic ITS2 secondary structures (Coleman 2003, Schultz et al. 2005). The region acquired from the field vegetative cells (WP187; accession number: ON065541) is highlighted in green.

**Table S1.** Primers used for amplification and sequencing of nuclear‐encoded small and large subunits of ribosomal DNA genes (SSU and LSU rDNA, respectively), internal transcribed spacer region 2 of nuclear rDNA (ITS2), RuBisCO large subunit gene (*rbc*L), ATP synthase beta subunit gene (*atp*B), and P700 chlorophyll a apoprotein A2 gene (*psa*B). This table is mainly based on Table S3 in Matsuzaki et al. (2015).

**Table S2.** Taxa and specimens/strains used for the molecular analyses (Figs. 3, S1, and S2) and DDBJ/ENA/GenBank accession numbers of their five genes. The asterisk indicates the authentic strain.

**Table S3.** List of meta‐amplicon sequencing datasets examined in this study screened for distribution hits of *Chloromonas kaweckae*.

**Table S4.** Comparison of vegetative cell and asexual reproduction characteristics of 12 snow‐inhabiting species of *Chloromonas* which have ovoid to ellipsoidal cell shapes without a prominent anterior papilla. This table is mainly based on Matsuzaki et al. (2018, 2019).
