# Supplementary material for: The snow alga Chloromonas kaweckae sp. nov. (Volvocales, Chlorophyta) causes green surface blooms in the high tatras (Slovakia) and tolerates high irradiance
Source: J Phycol. 2023 Jan 13;59(1):236–48. doi: 10.1111/jpy.13307 (PMC10946730; doi:10.1111/jpy.13307)
Supplement: Supplementary file 1 — Appendix S1. Key to vegetative cells of snow‐inhabiting species of Chloromonas sensu Ettl (1970, 1983). The key is mainly based on the previous key (Matsuzaki et al. 2018). Species examined using cultured material are marked with asterisks (on the basis of the supplemental references and the present study). [file JPY-59-236-s007.docx]

**Key to vegetative cells of snow-inhabiting species of *Chloromonas* sensu Ettl (1970, 1983).**

The key is mainly based on the previous key (Matsuzaki *et al.* 2018). Species examined using cultured material are marked with asterisks (on the basis of the supplemental references and the present study).

1. Cell inverted teardrop-shaped with a prominent posterior end ……………… *C. nivalis**

1. Cell not inverted teardrop-shaped with a prominent posterior end ………………………………………………………………………………………… 2

2. Cell not elongate or ellipsoidal ………………………………………………….3

2. Cell elongate or ellipsoidal ………………………………………………………… 6

3. Cell with prominent anterior papilla ………………………………………… 4

3. Cell without prominent anterior papilla ………………………………………… 5

4. Two contractile vacuoles positioned near the base of flagella; anterior papilla without concave top face; maximum cell length less than 15 μm ………………..*C. andreii*

4. Four contractile vacuoles positioned near the base of flagella; anterior papilla with concave top face; maximum cell length more than 15 μm ……………………*C. rubroleosa*

5. Cell ovoid or pyriform …………………………………………………… *C. brevispina*

5. Cell almost spherical ………………………………………………. *C. miwae**

6. Chloroplast asteroid-shaped; maximum cell length more than 40 μm

…………………………………………………………………. *C. bolyaiana*

6. Chloroplast not asteroid-shaped; maximum cell length less than 40 μm

……………………………………………………………………………… 7

7. Cell elongate kidney to elongate bean shaped ………………………………………… 8

7. Cell elongate cylindrical, elongate ellipsoidal, elongate ovoid or ellipsoidal ………………………………………………………………………………...12

8. Chloroplast cup-shaped ………………………………………………..…. *C. rostafinskii*

8. Chloroplast parietal on the dorsal side of the cell ………………………………… 9

9. Chloroplast with a longitudinal slit in the anterior half of the chloroplast; cell aggregates (more than 16 cells) formed in old cultures ……………...……..*C. polyptera**

9. Chloroplast without a longitudinal slit in the anterior half of the chloroplast; cell aggregates not formed in old cultures ……………………………………………… 10

10. Chloroplast filling in the posterior end of the protoplast …………… *C. krienitzii**

10. Chloroplast not filling in the posterior end of the protoplast ……………………… 11

11. Maximum cell width less than 10 μm; zoospores formed within the parental cell up to eight ……………………… ………………………………………… *C. fukushimae**

11. Maximum cell width more than 10 μm; zoospores formed within the parental cell generally up to four …………………………………………… *C. hindakii**

12. Chloroplast seemingly composed of elongate ovoid or elongate cylindrical platelets ……………………………………………………………………………… 13

12. Chloroplast seemingly composed of angular discs ……………………… 15

13. Cell aggregates formed in old cultures; zoospores formed within the parental cell up to 16 ……………………………………………………………… *C. tughillensis**

13. Cell aggregates not formed in old cultures; zoospores formed within the parental cell up to four or eight ……………………………………………………………………… 14

14. Cell elongate ellipsoidal; zoospores formed within the parental cell up to eight

………………..…………………………………………………… *C. hohamii**

14. Cell elongate cylindrical; zoospores formed within the parental cell up to four

……………………………………………………….……………… *C. tenuis**

15. Eyespot absent ………………………………………….…………………… 16

15. Eyespot present …………………………………………….………………… 18

16. Cell aggregates formed in old cultures …………………………….…… *C. pichinchae**

16. Cell aggregates not formed in old cultures ……………………………………… 17

17. Maximum cell length more than 20 μm; maximum cell width more than 13 μm …………………………………………………...… *C. chenangoensis**

17. Maximum cell length less than 20 μm; maximum cell width less than 13 μm ……………………………………………………………….…… *C. hoshawii**

18. Maximum cell length less than 15 μm ………………………..…….… *C. alpina*

18. Maximum cell length more than 15 μm ………………………………………….…… 19

19. Maximum cell length more than 25 μm; cell aggregates formed in old cultures

………………………………………………………………..…… *C. remiasii**

19. Maximum cell length less than 25 μm; cell aggregates not formed in old cultures

……………………………………………………………………………… 20

20. Zoospores formed within the parental cell up to eight; eyespot positioned in the anterior half to one third of the cell ………………………………… *C. muramotoi**

20. Zoospores formed within the parental cell up to four; eyespot positioned irregularly in the posterior third to anterior third of the cell ………………… *C. kaweckae* sp. nov.*

**References**

Ettl, H. 1970. Die Gattung *Chloromonas* Gobi emend. Wille (*Chlamydomonas* und die nächstverwandten gattungen I). *Nova Hedwigia Beih*. 34: 1–283.

Ettl, H. 1983. Chlorophyta 1. Phytomonadina. *In* Ettl, H., Gerloff, J., Heynig, H. & Mollenhauer, D. [Eds]. Süßwasserflora von Mitteleuropa 9. Stuttgart: G. Fischer Verlag. pp. 807.

Hoham, R.W., Bonome, T.A., Martin, C.W. & Leebens‐Mack, J. H. 2002. A combined 18S rDNA and *rbc*L phylogenetic analysis of *Chloromonas* and *Chlamydomonas* (Chlorophyceae, Volvocales) emphasizing snow and other cold-temperature habitats. *J*. *Phycol*. 38: 1051–1064.

Hoham. R.W., Berman. J.D., Rogers, H.S., Felio, J.H., Ryba, J.B. & Miller, P.R. 2006. Two new species of green snow algae from Upstate New York, *Chloromonas chenangoensis* sp. nov. and *Chloromonas tughillensis* sp. nov. (Volvocales, Chlorophyceae) and the effects of light on their life cycle development. *Phycologia* 45: 319–330.

Kirjakov, I.K. & Velichkova, K.N. 2016. New species of green snow algae *Chloromonas* (Volvocales, Chlorophyta) from Bulgaria. *Int*. *J*. *Fis*. *Stu*. 4: 94–95.

Ling, H.U. & Seppelt, R.D. 1993. Snow algae of the Windmill Islands, continental Antarctica. 2. *Chloromonas rubroleosa* sp. nov. (Volvocales, Chlorophyta), an alga of red snow. *Eur*. *J*. *Phycol*. 28: 77–84.

Matsuzaki, R., Hara, Y. & Nozaki, H. 2014. A taxonomic study of snow *Chloromonas* species (Volvocales, Chlorophyceae) based on light and electron microscopy and molecular analysis of cultured material. *Phycologia* 53: 293–304.

Matsuzaki, R., Kawai-Toyooka, H., Hara, Y. & Nozaki, H. 2015. Revisiting the taxonomic significance of aplanozygote morphologies of two cosmopolitan snow species of the genus *Chloromonas* (Volvocales, Chlorophyceae). *Phycologia* 54: 491–502.

Matsuzaki, R., Nozaki, H. & Kawachi, M. 2018. Taxonomic revision of *Chloromonas nivalis* (Volvocales, Chlorophyceae) strains, with the new description of two snow-inhabiting *Chloromonas* species. *PLoS One* 13: e0193603.

Matsuzaki, R., Nozaki, H., Takeuchi, N., Hara, Y. & Kawachi, M. 2019. Taxonomic re-examination of “*Chloromonas nivalis* (Volvocales, Chlorophyceae) zygotes” from Japan and description of *C. muramotoi* sp. nov. *PLoS One* 14: e0210986.

Muramoto, K., Nakada, T., Shitara, T., Hara, Y. & Nozaki, H. 2010. Re-examination of the snow algal species *Chloromonas miwae* (Fukushima) Muramoto et al., comb. nov. (Volvocales, Chlorophyceae) from Japan, based on molecular phylogeny and cultured material. *Eur*. *J*. *Phycol*. 45: 27–37.

Procházková, L., Remias, D., Řezanka, T. & Nedbalová, L. 2019. Ecophysiology of *Chloromonas hindakii* sp. nov. (Chlorophyceae), causing orange snow blooms at different light conditions. *Microorganisms* 7: 434.
