# Supplementary figures and images for: The snow alga Chloromonas kaweckae sp. nov. (Volvocales, Chlorophyta) causes green surface blooms in the high tatras (Slovakia) and tolerates high irradiance
Source: J Phycol. 2023 Jan 13;59(1):236–48. doi: 10.1111/jpy.13307 (PMC10946730; doi:10.1111/jpy.13307)

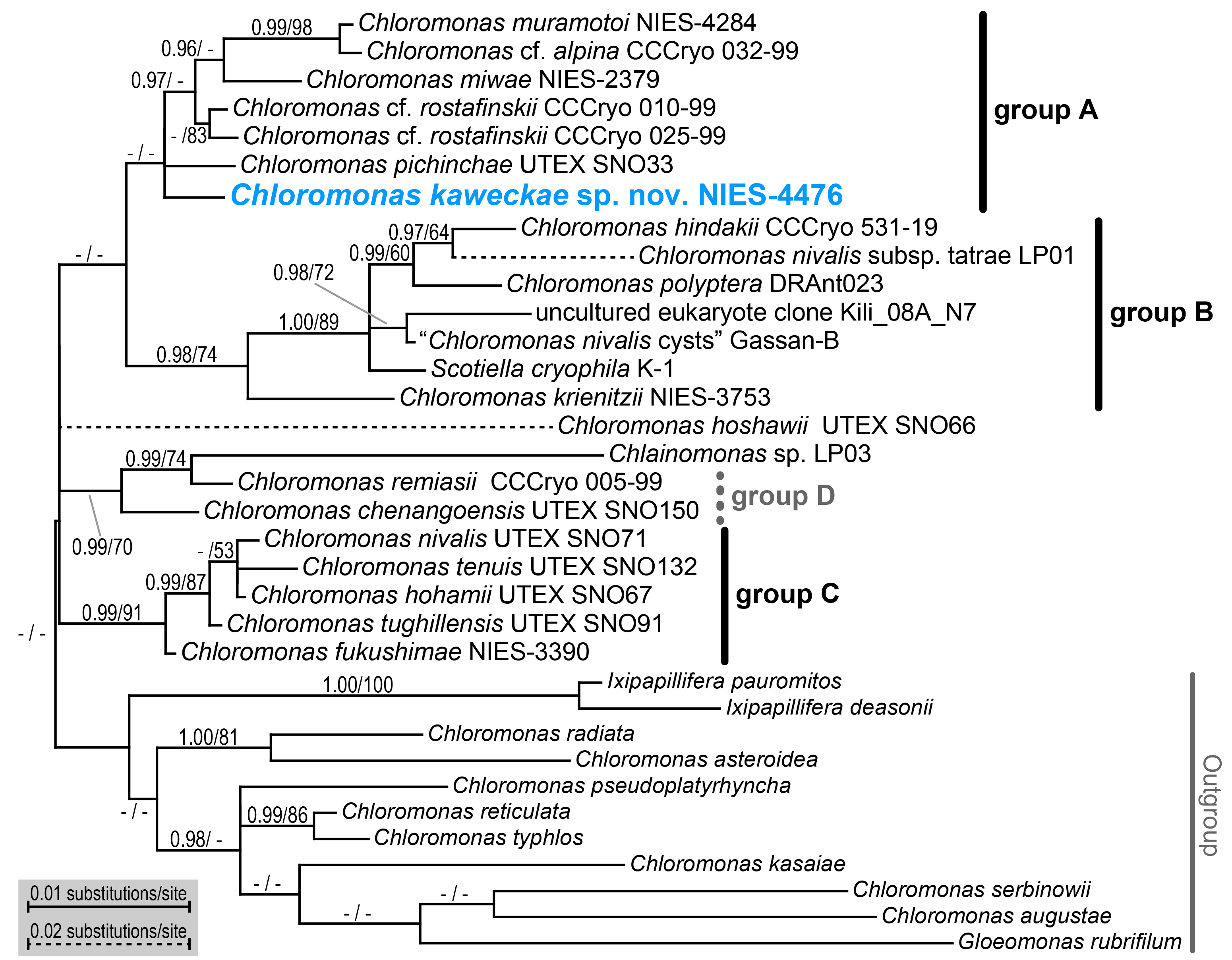

Supplement: Supplementary file 2 — Figure S1. Bayesian phylogenetic tree of snow inhabiting Chloromonas spp. based on 1,621 bp of nuclear encoded SSU ribosomal DNA (18S rDNA gene). The applied substitution model was K80 + I + G4. Clade names are indicated according to Matsuzaki et al. (2019). The corresponding posterior probabilities of Bayesian inference (0.90 or more, left) and bootstrap values from a maximum likelihood analysis (50% or more, right) are shown at each node (Matsuzaki et al. 2019). [file JPY-59-236-s002.tif]

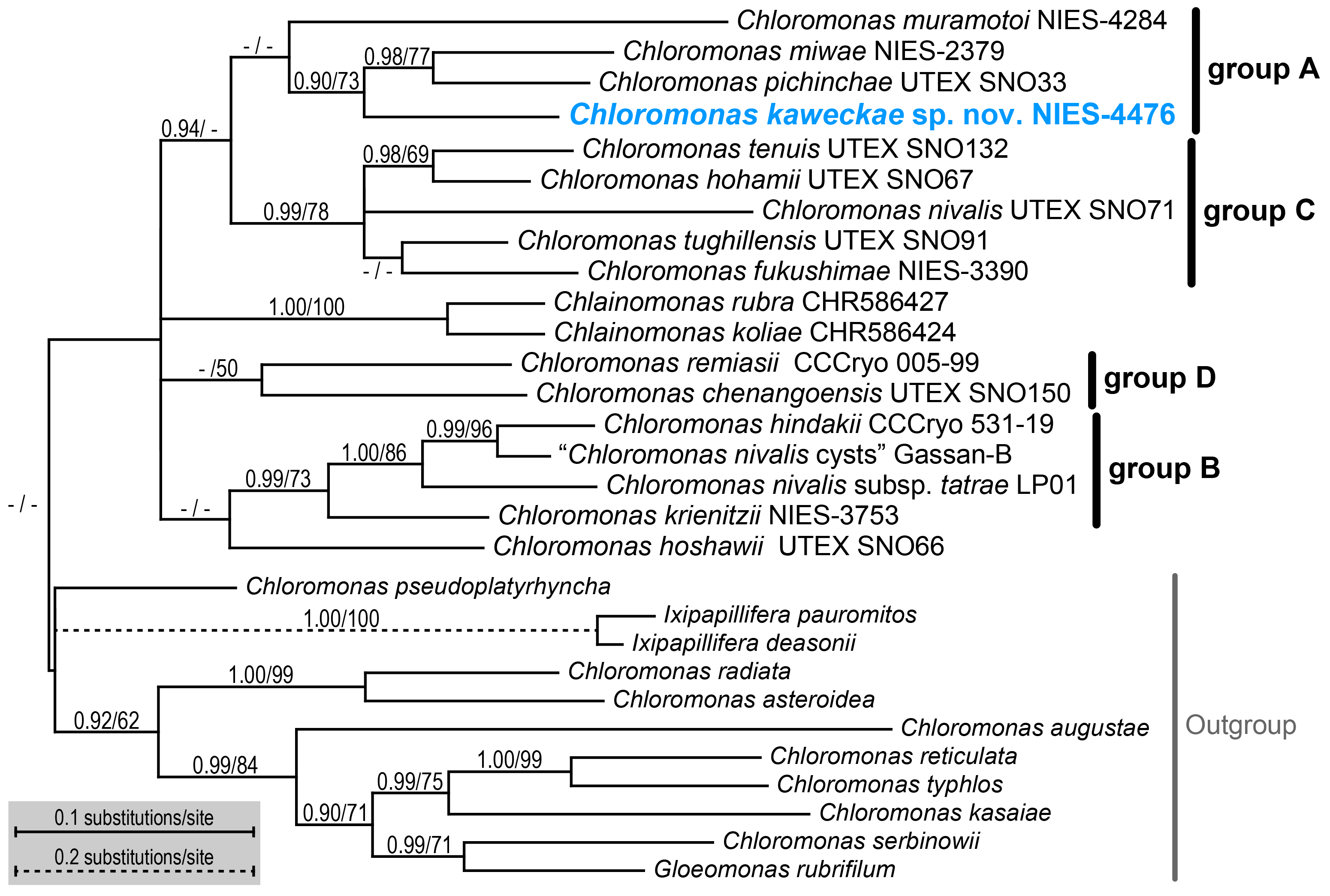

Supplement: Supplementary file 3 — Figure S2. Bayesian phylogenetic tree based on 1,128 bp of the rbcL gene. The applied substitution models are GTR + I + G4 for the first and third codon positions and JC + I for the second codon position. Clade names are indicated according to Matsuzaki et al. (2019). The corresponding posterior probabilities of Bayesian inference (0.90 or more, left) and bootstrap values from a maximum likelihood analysis (50% or more, right) are shown at each node. [file JPY-59-236-s001.tif]

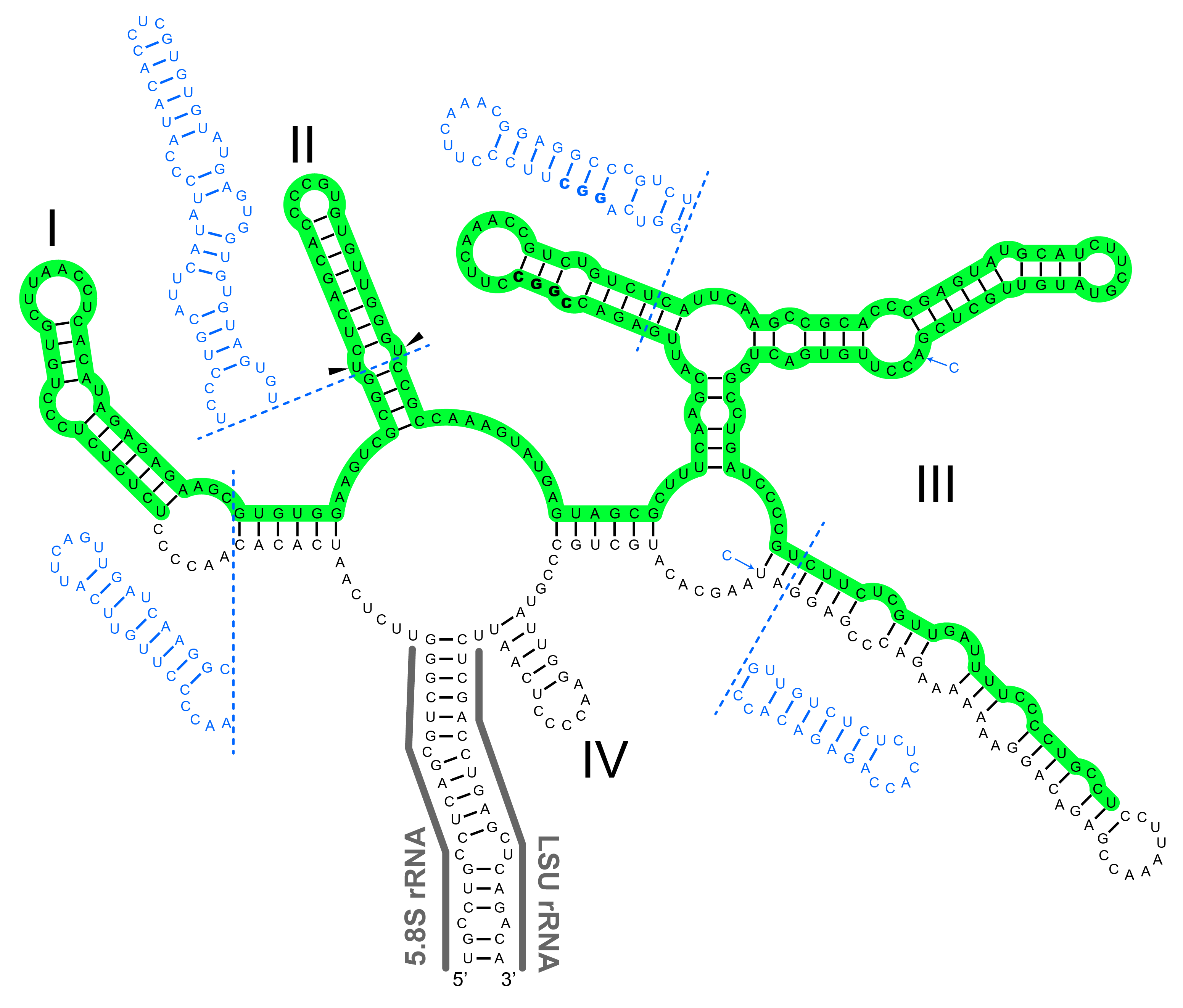

Supplement: Supplementary file 4 — Figure S3. Secondary structure of ITS2 transcript of Chloromonas kaweckae NIES‐4476 (accession number: LC683783). The 3′ end of the 5.8 S rRNA and the 5′ end of the LSU rRNA are shown. Differences between the strain and Chloromonas pichinchae strain UTEX SNO33 (accession number: LC012761) are described just outside the structure as blue characters. Note U–U mismatch in helix II (arrowheads) and the YGGY motif on the 5′side near the apex of helix III (boldface), common structural hallmarks of eukaryotic ITS2 secondary structures (Coleman 2003, Schultz et al. 2005). The region acquired from the field vegetative cells (WP187; accession number: ON065541) is highlighted in green. [file JPY-59-236-s008.tif]
