# Supplementary material for: The snow alga Chloromonas kaweckae sp. nov. (Volvocales, Chlorophyta) causes green surface blooms in the high tatras (Slovakia) and tolerates high irradiance
Source: J Phycol. 2023 Jan 13;59(1):236–48. doi: 10.1111/jpy.13307 (PMC10946730; doi:10.1111/jpy.13307)
Supplement: Supplementary file 5 — Table S1. Primers used for amplification and sequencing of nuclear‐encoded small and large subunits of ribosomal DNA genes (SSU and LSU rDNA, respectively), internal transcribed spacer region 2 of nuclear rDNA (ITS2), RuBisCO large subunit gene (rbcL), ATP synthase beta subunit gene (atpB), and P700 chlorophyll a apoprotein A2 gene (psaB). This table is mainly based on Table S3 in Matsuzaki et al. (2015). [file JPY-59-236-s004.docx]

**Table S1.** Primers used for amplifications and sequencing of nuclear-encoded small and large subunits of ribosomal DNA (SSU and LSU rDNA, respectively), internal transcribed spacer region 2 of nuclear rDNA (ITS2), RuBisCO large subunit gene (*rbc*L), ATP synthase beta subunit gene (*atp*B), and P700 chlorophyll *a* apoprotein A2 gene (*psa*B). This table is mainly based on the table S3 in Matsuzaki et al. (2015).

| **Designation** | **Position^1^** | **Sequence (5’–3’)** |
| --- | --- | --- |
| **SSU rDNA** |  |  |
| FA^2^ | 1–21 | AACCTGGTTGATCCTGCCAGT |
| FC^3^ | 458–478 | GGGAGGTAGTGACAAIAAATA |
| RD^2^ | 570–550^4^ | GCTGGCACCAGACTTGCCCTC |
| FE^2^ | 1112–1132 | GGGAGTATGGTCGCAAGGCTG |
| RF^2^ | 1202–1182^4^ | CCCGTGTTGAGTCAAATTAAG |
| RB^2^ | 1799–1774^4^ | TGATCCTTCTGCAGGTTCACCTAC |
| **LSU rDNA** |  |  |
| F1^5^ | 77–101 | ATTCCCCTAGTAACGGCGAGCGAAC |
| F2^5^ | 804–826 | CGAAAGATGGTGAACTATGCCTG |
| R1^5^ | 882–860^4^ | TGCACGTCAGCACATCTACGAGC |
| F3^5^ | 1501–1521 | CAGCAATTGGACATGGGTTAG |
| R2^5^ | 1726–1702^4^ | CTATCGCCGGATGATCCGATTCCAG |
| R3^5^ | 2159–2139^4^ | CCTTTAGGACCATCACAATGC |
| **ITS2** |  |  |
| Fa^6^ | 1761–1787^7^ | GGGATCCGTTTCCGTAGGTGAACCTGC |
| Fc^6^ | 30–49^8^ | GCATCGATGAAGAACGCAGC |
| Rb^6^ | 50–23^4, 9^ | GGGATCCATATGCTTAAGTTCAGCGGGT |
| ***rbc*L** |  |  |
| F1^10^ | 1–20 | ATGGTTCCACAAACAGAAAC |
| Snow-F1^11^ | 178–200 | GAATCTTCWACWGGTACTTGGAC |
| Snow-F4^12^ | 472–494 | GAACGTGACAAATTAAACAAATA |
| Snow-R3^11^ | 656–634^4^ | ATRAAACGGTCTCTCCAACGCAT |
| Snow-R12^12^ | 1030–1006^4^ | CTAAAGTAACTTCACGTTCTCCTTC |
| R42AE^13^ | 1403–1384^4^ | TCRAAYTTRATYTCYTTCCA |
| R3^10^ | 1421–1402^4^ | TTGTCAATAGTATCAAATTC |
| ***atp*B** |  |  |
| F1^14^ | 180–200 | TGTTACTTGTGAAGTTCAACA |
| F30^15^ | 208–227 | GGTGATAAYTGTGTAMGWGC |
| F2^14^ | 721–741 | GAACCACCAGGTGCTCGTATG |
| R3^14^ | 895–875^4^ | GGTAACCTACAGCTGATGGCA |
| R4^14^ | 1433–1412^4^ | CCTACTAAGTAGAATGCTTGTT |
| ***psa*B** |  |  |
| F1^16^ | 205–224 | GCITGGCARGGIAAYTTYGA |
| F5^16^ | 989–1010 | TACAYTTCCAATTAGGYTTAGC |
| R2^16^ | 1133–1114^4^ | ATRTAYTGRTGRTGIGTRTA |
| R6^16^ | 1760–1741^4^ | ATIGTRTTIARCATCCARAA |

^1^Coordinate numbers from SSU rDNA of *Chlorella vulgaris* (Huss and Sogin 1990), LSU rDNA of *Oryza sativa* (Takaiwa et al. 1985), the nuclear-encoded 5.8S or LSU rDNA of *Didymogenes soliella* (Hoshina and Fujiwara 2013), *rbcL* of *Chlorella ellipsoidea* (Yoshinaga et al. 1988), or *atp*B or *psa*B of *Chlorella vulgaris* (Wakasugi et al. 1997), respectively.

^2^Primer sourced from Nakazawa and Nozaki (2004).

^3^Primer sourced from Nakada et al. (2007).

^4^Reverse primer.

^5^Primer sourced from Matsuzaki et al. (2014)

^6^Primer sourced from Coleman (1994).

^7^Position in SSU rDNA.

^8^Position in 5.8S rDNA.

^9^Position in LSU rDNA.

^10^Primer sourced from Nozaki et al. (1995).

^11^Primer sourced from Muramoto et al. (2008).

^12^Primer sourced from Matsuzaki et al. (2015).

^13^Primer sourced from Shimada et al. (1995).

^14^Primer sourced from Nozaki et al. (1999).

^15^Primer sourced from Nozaki et al. (2002).

^16^Primer sourced from Nozaki et al. (2000).

**References**

Coleman, A.W. 1994. Molecular delineation of species and syngens in Volvocacean green algae (Chlorophyta). *J. Phycol.* 30: 30–90.

Hoshina, R. & Fujiwara, Y. 2013. Molecular characterization of *Chlorella* cultures of the National Institute for Environmental Studies culture collection with description of *Micractinium inermum* sp. nov., *Didymogenes sphaerica* sp. nov., and *Didymogenes soliella* sp. nov. (Chlorellaceae, Trebouxiophyceae). *Phycol. Res.* 61: 124–132.

Huss, V.A.R. & Sogin, M.L. 1990. Phylogenetic position of some *Chlorella* species within the Chlorococcales based upon complete small-subunit ribosomal RNA sequences. *J. Mol. Evol.* 31: 432–442.

Matsuzaki, R., Hara, Y. & Nozaki, H. 2014. A taxonomic study of snow *Chloromonas* species (Volvocales, Chlorophyceae) based on light and electron microscopy and molecular analysis of cultured material. *Phycologia* 35: 293–304.

Matsuzaki, R., Kawai-Toyooka, H., Hara, Y. & Nozaki, H. 2015. Revisiting the taxonomic significance of aplanozygote morphologies of two cosmopolitan snow species of the genus *Chloromonas* (Volvocales, Chlorophyceae). *Phycologia* 54: 491–502.

Muramoto, K., Kato, S., Shitara, T., Hara, Y. & Nozaki, H. 2008. Morphological and genetic variation in the cosmopolitan snow alga *Chloromonas nivalis* (Volvocales, Chlorophyta) from Japanese mountainous area. *Cytologia* 73: 91–96.

Nakada, T., Suda, S. & Nozaki, H. 2007. A taxonomic study of *Hafniomonas* (Chlorophyceae) based on a comparative examination of cultured material. *J. Phycol.* 43: 397–411.

Nakazawa, A. & Nozaki, H. 2004. Phylogenetic analysis of the tetrasporalean genus *Asterococcus* (Chlorophyceae) based on 18S ribosomal RNA gene sequences. *J. Jpn. Bot.* 79: 255–261.

Nozaki, H., Itoh, M., Sano, R., Uchida, H., Watanabe, M.M. & Kuroiwa, T. 1995. Phylogenetic relationships within the colonial Volvocales (Chlorophyta) inferred from *rbc*L gene sequence data. *J. Phycol.* 31: 970–979.

Nozaki, H., Ohta, N., Takano, H. & Watanabe, M.M. 1999. Reexamination of phylogenetic relationships within the colonial Volvocales (Chlorophyta): an analysis of *atp*B and *rbc*L gene sequences. *J. Phycol.* 35: 104–112.

Nozaki, H., Misawa, K., Kajita, T., Kato, M., Nohara, S. & Watanabe, M.M. 2000. Origin and evolution of the colonial Volvocales (Chlorophyceae) as inferred from multiple, chloroplast gene sequences. *Mol. Phylogenet. Evol.* 17: 256–268.

Nozaki, H., Onishi, K. & Morita, E. 2002. Differences in pyrenoid morphology are correlated with differences in the *rbc*L genes of members of the *Chloromonas* lineage (Volvocales, Chlorophyceae). *J. Mol. Evol.* 55: 414–430.

Shimada, A., Kanai, S. & Maruyama, T. 1995. Partial sequence of ribulose-1,5-bisphosphate carboxylase/oxygenase and the phylogeny of *Prochloron* and *Prochlorococcus* (Prochlorales). *J. Mol. Evol.* 40: 671–677.

Takaiwa, F., Oono, K., Iida, Y. & Sugiura, M. 1985. The complete nucleotide sequence of a rice 25S·rRNA gene. *Gene* 37: 255–259.

Wakasugi, T., Nagai, T., Kapoor, M., Sugita, M., Ito, M., Ito, S., Tsudzuki, J., Nakashima, K., Tsudzuki, T., Suzuki, Y., Hamada, A., Ohta, T., Inamura, A., Yoshinaga, K. & Sugiura, M. 1997. Complete nucleotide sequence of the chloroplast genome from the green alga *Chlorella vulgaris*: The existence of genes possibly involved in chloroplast division. *P. Natl. Acad. Sci. USA* 94: 5967–5972.

Yoshinaga, K., Ohta, T., Suzuki, Y. & Sugiura, M. 1988. *Chlorella* chloroplast DNA sequence containing a gene for the large subunit of ribulose-1,5-biphosphate carboxylase/oxygenase and a part of a possible gene for the beta’ subunit of RNA polymerase. *Plant Mol. Biol.* 10: 245–250.
