# Supplementary material for: The snow alga Chloromonas kaweckae sp. nov. (Volvocales, Chlorophyta) causes green surface blooms in the high tatras (Slovakia) and tolerates high irradiance
Source: J Phycol. 2023 Jan 13;59(1):236–48. doi: 10.1111/jpy.13307 (PMC10946730; doi:10.1111/jpy.13307)
Supplement: Supplementary file 6 — Table S2. Taxa and specimens/strains used for the molecular analyses (Figs. 3, S1, and S2) and DDBJ/ENA/GenBank accession numbers of their five genes. The asterisk indicates the authentic strain. [file JPY-59-236-s006.docx]

**Table S2.** Taxa and specimens/strains used for the molecular analyses (Figs 3–5, Figs S1–S3 in the Supporting information) and DDBJ/ENA/GenBank accession numbers of their five genes. Asterisk indicates the authentic strain.

| Taxon | Specimen/strain designation | Accession number | | | | |
| --- | --- | --- | --- | --- | --- | --- |
|  |  | SSU rDNA | LSU rDNA | *rbc*L | *atp*B | *psa*B |
| Ingroup | | | | | | |
| *Cr. chenangoensis* | UTEX^1^ SNO150* | AB906341 | LC360468 | LC012736 | AB906360 | AB906371 |
| *Cr. fukushimae* | NIES^2^-3390 | AB906343 | AB906353 | LC012739 | AB906362 | AB906373 |
| *Cr. hindakii* | CCCryo^3^ 531-19* | MN251865 |  | MN251877.2 |  |  |
| *Cr. hohamii* | UTEX SNO67 | AB906344 | AB906354 | AB434265,  LC012742 | AB906363 | AB906374 |
| *Cr. hoshawii* | UTEX SNO66* | AB906345 | LC360469 | AB434272 | AB906364 | AB906375 |
| *Cr. kaweckae* sp. nov. | NIES-4476 | LC683781^4^ | LC683782^4^ | LC683784^4^ | LC683785^4^ | LC683786^4^ |
| *Cr. krienitzii* | NIES-3753* | LC012712 | LC060474 | LC012740 | LC012720 | LC012728 |
| *Cr. miwae* | NIES-2379 | AB906350 | LC060476 | AB434271 | AB906369 | AB906380 |
| *Cr. muramotoi* | NIEX-4284* | LC438435 | LC438439 | LC438451 | LC438443 | LC438447 |
| *Cr. nivalis* | UTEX SNO71 | LC360465 | LC360470 | LC360492 | LC360484 | LC360488 |
|  | Gassan-B^5^ | LC012714 | LC060478 | LC012743 | LC012722 | LC012730 |
| *Cr. nivalis* subsp. *tatrae* | LP01^6^ | KY499614 |  | KY499615.2 |  |  |
| *Cr. pichinchae* | UTEX SNO33 | AB906346 | LC060481 | AB434266,  LC012746 | AB906365 | AB906376 |
| *Cr. polyptera* | DRAnt023^7^ | JQ790556 |  |  |  |  |
| *Cr. remiasii* | CCCryo 005-99* | LC360466 | LC360471 | LC360493 | LC360485 | LC360489 |
| *Cr. tenuis* | UTEX SNO132* | AB906347 | AB906355 | AB434263 | AB906366 | AB906377 |
| *Cr. tughillensis* | UTEX SNO91* | AB906348 | AB906356 | LC012747 | AB906367 | AB906378 |
| *Cr.* cf. *alpina* | CCCryo 032-99 | AF514403 |  |  |  |  |
| *Cr.* cf. *rostafinskii* | CCCryo 010-99 | AF514400 |  |  |  |  |
|  | CCCryo 025-99 | AF514402 |  |  |  |  |
| *Ci. koliae* | CHR586424^8^ |  |  | DQ885962 |  |  |
| *Ci. rubra* | CHR586427^9^ |  |  | DQ885969 |  |  |
| *Ci.* sp. | LP03^10^ | MF803745 |  |  |  |  |
| *Sc. cryophila* | K-1^11^ | MG253843 |  |  |  |  |
| uncultured clone | Kili_08A_N7^12^ | KX771778 |  |  |  |  |
| Outgroup | | | | | | |
| *Cr. asteroidea* | SAG^13^ 11-47^14^ | U70783 | LC360473 | AB022225 | AB084808 | AB084342 |
| *Cr. augustae* | SAG 5.73^14^ | AJ410452 | LC360474 | AB504764 | AB504757 | AB504769 |
| *Cr. chlorococcoides* | SAG 15.82* | AJ410449,  AB624555 | AB906359 | LC361432 | AB624580 | AB624595 |
| *Cr. kasaiae* | NIES-2862* | AB734109 | LC360475 | LC012751 | AB734110 | AB734111 |
| *Cr. pseudoplatyrhyncha* | NIES-2563 | AB548689 | LC360476 | LC012752 | AB548690 | AB548691 |
| *Cr. radiata* | UTEX 966^14^ | U57697 | LC360477 | AJ001878 | AB084311 | AB084345 |
| *Cr. reticulata* | SAG 29.83  (= UTEX 1970^14^) | U70791,  AB624560 | AF395508 | AB022534 | AB084312 | AB084346, AB084347 |
| *Cr. serbinowii* | UTEX 492^14^  (= SAG 11.84) | U70795, AB624568, AB624569 | LC360478 | AJ001879 | AB084317 | AB084354 |
| *Cr. typhlos* | SAG 26.86  (= UTEX 1969) | AB624566 | LC360479 | AB022228 | AB084307 | AB084341 |
| *Gl. rubrifilum* | SAG 3.85^15^ | AJ410455 | LC360481 | AB504765 | AB504758 | AB504770 |
| *Ix. deasonii* | SAG 46.72* | AJ410446 | LC360482 | AB101508 | AB101503 | AB101514 |
| *Ix. pauromitos* | NIES-3707* | LC057290 | LC360483 | LC360495 | LC360487 | LC360491 |

Abbreviations: *atp*B, ATP synthase beta subunit gene; *Ci.*, *Chlainomonas*; *Cr.*, *Chloromonas*; *Gl.*, *Gloeomonas*; *Ix.*, *Ixipapillifera*; LSU rDNA, large subunit ribosomal DNA; *psa*B, P700 chlorophyll *a* apoprotein A2 gene; *rbc*L, RuBisCO large subunit gene; *Sc.*, *Scotiella*; SSU rDNA, small subunit ribosomal DNA.

^1^Culture Collection of Algae at the University of Texas at Austin, USA (<https://utex.org/>).

^2^Microbial Culture Collection at the National Institute for Environmental Studies, Japan (<https://mcc.nies.go.jp/index_en.html>).

^3^Culture Collection of Cryophilic Algae at the Fraunhofer Institute for Cell Therapy and Immunology, Germany (<http://cccryo.fraunhofer.de/web/infos/welcome/>).

^4^Sequenced in the present study.

^5^Specimen of cysts collected from snowpack on Mt. Gassan, Japan (Matsuzaki *et al.* 2015).

^6^Specimen of cysts collected from snowpack on the High Tatras, Slovakia (Procházková *et al.* 2018a).

^7^Specimen of cysts collected from snowpack on Antarctic Peninsula (Remias *et al.* 2013).

^8^Specimen of vegetative cells collected from snowpack on Ahuriri Valley, New Zealand (Novis *et al.* 2008).

^9^Specimen of vegetative cells collected from snowpack on Cle Elum Valley, USA (Novis *et al.* 2008).

^10^Specimen of vegetative cells collected from snowpack on the High Tatras, Slovakia (Procházková *et al.* 2018b).

^11^Specimen of cysts collected from snowpack on Austrian Alps (Remias *et al.* 2018).

^12^Environmental sequence originating from a soil sample from Mt. Kilimanjaro, Tanzania (Vimercati *et al.* 2019).

^13^Sammlung von Algenkulturen at the University of Göttingen (<http://sagdb.uni-goettingen.de/>).

^14^Epitype proposed by Pröschold *et al.* (2001).

^15^Epitype of *Chloromonas rubrifilum* designated by Pröschold *et al.* (2001).

**References**

Matsuzaki, R., Kawai-Toyooka, H., Hara, Y. & Nozaki, H. 2015. Revisiting the taxonomic significance of aplanozygote morphologies of two cosmopolitan snow species of the genus *Chloromonas* (Volvocales, Chlorophyceae). *Phycologia* 54: 491–502.

Novis, P.M.., Hoham, R.W., Beer, T. & Dawson, M. 2008. Two snow species of the quadriflagellate green alga *Chlainomonas* (Chlorophyta, Volvocales): ultrastructure and phylogenetic position within the Chloromonas clade. *J. Phycol.* 44: 1001–1012.

Procházková, L., Remias, D., Holzinger, A., Řezanka, T. & Nedbalová, L. 2018b. Ecophysiological and morphological comparison of two populations of *Chlainomonas* sp. (Chlorophyta) causing red snow on ice-covered lakes in the High Tatras and Austrian Alps. *Eur. J. Phycol.* 53: 230–243.

Procházková, L., Remias, D., Řezanka, T. & Nedbalová, L. 2018a. *Chloromonas nivalis* subsp. *tatrae*, subsp. nov. (Chlamydomonadales, Chlorophyta): re-examination of a snow alga from the High Tatra Mountains (Slovakia). *Fottea* 18: 1–18.

Pröschold, T., Marin, B., Schlösser, UG. & Melkonian, M. 2001. Molecular phylogeny and taxonomic revision of *Chlamydomonas* (Chlorophyta). I. Emendation of *Chlamydomonas* Ehrenberg and *Chloromonas* Gobi, and description of *Oogamochlamys* gen. nov. and *Lobochlamys* gen. nov. *Protist* 152: 265–300.

Remias, D., Wastian, H., Lütz, C. & Leya, T. 2013. Insights into the biology and physiology of *Chloromonas polyptera* (Chlorophyta), an alga causing orange snow in Maritime Antarctica. *Antarct. Sci.* 25: 648–656.

Remias, D., Procházková, L., Holzinger, A. & Nedbalova, L. 2018. Ecology, cytology and phylogeny of the snow alga *Scotiella cryophila* K-1 (Chlamydomonadales, Chlorophyta) from the Austrian Alps. *Phycologia* 57: 581–592.

Vimercati, L., Darcy, J.L. & Schmidt, S.K. 2019. The disappearing periglacial ecosystem atop Mt. Kilimanjaro supports both cosmopolitan and endemic microbial communities. *Sci. Rep.* 9: 10676.
