# Supplementary material for: The snow alga Chloromonas kaweckae sp. nov. (Volvocales, Chlorophyta) causes green surface blooms in the high tatras (Slovakia) and tolerates high irradiance
Source: J Phycol. 2023 Jan 13;59(1):236–48. doi: 10.1111/jpy.13307 (PMC10946730; doi:10.1111/jpy.13307)
Supplement: Supplementary file 8 — Table S4. Comparison of vegetative cell and asexual reproduction characteristics of 12 snow‐inhabiting species of Chloromonas which have ovoid to ellipsoidal cell shapes without a prominent anterior papilla. This table is mainly based on Matsuzaki et al. (2018, 2019). [file JPY-59-236-s003.docx]

**Table S4.** Comparison of vegetative cell and asexual reproduction characteristics of 12 snow-inhabiting species of *Chloromonas* which have ovoid to ellipsoidal cell shapes without a prominent anterior papilla. This table is mainly based on Matsuzaki *et al.* (2018, 2019).

|  | *C. kaweckae* sp. nov. | *C. alpina* | *C. bolyaiana* | *C. brevispina* | *C. chenangoensis* |
| --- | --- | --- | --- | --- | --- |
| Strain(s) examined | NIES-4476, CCCryo 557-22 | − | − | − | UTEX SNO150, UTEX SNO143 |
| Cell shape | ovoid, elongate-ovoid to ellipsoidal | ellipsoidal to ovoid | ellipsoidal | ellipsoidal to ovoid or pyriform | ellipsoidal |
| Cell width × cell length (μm) | 9.2–15.5 × 16.0–22.5 | 4–7 × 9–12 | 46 – 52 × 52 – 72 | 5–13 × 10–19 | 7.5–17.5 × 14–25 |
| Chloroplast shape | cup-shaped with irregular incisions; seemingly composed of angular discs | parietal, seemingly composed of numerous discoid lobes obviously separated from each other | asteroid-shaped | cup-shaped | cup-shaped, seemingly composed of angular discs |
| Eyespot | generally ellipsoidal but variable, positioned in the posterior third to anterior third of the cell | ellipsoidal, positioned anterior third of the cell | absent | absent | absent |
| Cell aggregates in old culture | not observed | − | − | − | not observed |
| Number of zoospores formed within the parental cell wall | 2 or 4 | 2 or 4 | 2, 4 or 8 | up to 8 | 2 or 4 |
| References | present study | Wille (1903) | Kol (1947) | Hoham et al. (1979) | Hoham et al. (2006); Matsuzaki et al. (2014) |

**Table S4.** Extended

| *C. hohamii* | *C. hoshawii* | *C. miwae* | *C. muramotoi* | *C. pichinchae* | *C. remiasii* | *C. tughillensis* |
| --- | --- | --- | --- | --- | --- | --- |
| UTEX SNO67 | UTEX SNO66 | NIES-2379, NIES-2380 | NIES-4284 | UTEX SNO33 | CCCryo 005-99, CCCryo 047-99 | UTEX SNO91, UTEX SNO88, UTEX SNO92 |
| elongate-ellipsoidal | ellipsoidal to elongate-ovoid | spherical or ovoid | ovoid or spindle-shaped with a rounded posterior end | elongate-ovoid to ellipsoidal | ellipsoidal to spindle-shaped | elongate-ellipsoidal |
| 5–10 × 12–25 | 4.9–9.3 × 13.8–18.6 | 10–13 × 9–15 | 8.5–13.3 × 12.3–19.5 | 8–15 × 18–26 | 10.2–15.6 × 18.2–30.8 | 6–12 × 16–23 |
| cup-shaped, apparently composed of elongate-ovoid or elongate-cylindrical platelets | cup-shaped, seemingly composed of angular discs | cup-shaped, seemingly composed of angular discs | cup-shaped, seemingly composed of angular discs | cup-shaped, seemingly composed of angular discs | Cup-shaped, seemingly composed of angular discs | Cup-shaped, apparently composed of elongate-ovoid or elongate-cylindrical platelets |
| absent or generally present | absent | absent | D-shaped to rod-shaped, positioned in the anterior half to one third of the cell | absent | ellipsoidal to elongate D-shaped, positioned in the anterior third of the cell | not observed |
| not observed | not observed | − | not observed | observed | observed | observed |
| 2, 4 or 8 | 2 or 4 (rarely 8) | generally 2 or 4 | up to 8 | generally 2 or 4 | 2 or 4 (rarely 8) | 2, 4, 8 or 16 |
| Hoham et al. (1983); Matsuzaki et al. (2014) | Matsuzaki et al. (2018) | Muramoto et al. (2010) | Matsuzaki et al. (2019) | Wille (1903); Hoham (1975); Matsuzaki et al. (2014) | Matsuzaki et al. (2018) | Hoham et al. (2006); Matsuzaki et al. (2014) |

**References**

Ettl, H. 1970. Die Gattung *Chloromonas* Gobi emend. Wille (*Chlamydomonas* und die nächstverwandten gattungen I). *Nova Hedwigia Beih*. 34: 1–283.

Ettl, H. 1983. Chlorophyta 1. Phytomonadina. *In* Ettl, H., Gerloff, J., Heynig, H. & Mollenhauer, D. [Eds]. Süßwasserflora von Mitteleuropa 9. Stuttgart: G. Fischer Verlag. pp. 807.

Hoham, R. W. 1975. The life history and ecology of the snow alga *Chloromonas pichinchae* (Chlorophyta, Volvocales). *Phycologia* 14: 213–226.

Hoham, R. W., Roemer, S. C. & Mullet, J. E. 1979. The life history and ecology of the snow alga *Chloromonas brevispina* comb. nov. (Chlorophyta, Volvocales). *Phycologia* 18: 55–70.

Hoham, R. W., Mullet, J. E. & Roemer, S. C. 1983. The life history and ecology of the snow alga *Chloromonas polyptera* comb. nov. (Chlorophyta, Volvocales). *Phycologia* 61: 2416–2429.

Hoham. R.W., Berman. J.D., Rogers, H.S., Felio, J.H., Ryba, J.B. & Miller, P.R. 2006. Two new species of green snow algae from Upstate New York, *Chloromonas chenangoensis* sp. nov. and *Chloromonas tughillensis* sp. nov. (Volvocales, Chlorophyceae) and the effects of light on their life cycle development. *Phycologia* 45: 319–330.

Kol E. 1947. A new cryobiont of the red snow from Transylvania: *Chlamydomonas bolyaiana* n. sp. *Acta Bolyaiana* 1: 132–137.

Matsuzaki, R., Hara, Y. & Nozaki, H. 2014. A taxonomic study of snow *Chloromonas* species (Volvocales, Chlorophyceae) based on light and electron microscopy and molecular analysis of cultured material. *Phycologia* 53: 293–304.

Matsuzaki, R., Nozaki, H. & Kawachi, M. 2018. Taxonomic revision of *Chloromonas nivalis* (Volvocales, Chlorophyceae) strains, with the new description of two snow-inhabiting *Chloromonas* species. *PLoS One* 13: e0193603.

Matsuzaki, R., Nozaki, H., Takeuchi, N., Hara, Y. & Kawachi, M. 2019. Taxonomic re-examination of “*Chloromonas nivalis* (Volvocales, Chlorophyceae) zygotes” from Japan and description of *C. muramotoi* sp. nov. *PLoS One* 14: e0210986.

Muramoto, K., Nakada, T., Shitara, T., Hara, Y. & Nozaki, H. 2010. Re-examination of the snow algal species *Chloromonas miwae* (Fukushima) Muramoto et al., comb. nov. (Volvocales, Chlorophyceae) from Japan, based on molecular phylogeny and cultured material. *Eur*. *J*. *Phycol*. 45: 27–37.

Wille, N. 1903. Algologische Notizen. *Algologische Notizen IX–XIV. Nyt Magazin for Naturvidenskaberne* 41: 89–185.
